# Rescue of spinal muscular atrophy mouse models with AAV9-Exon-specific U1 snRNA

**DOI:** 10.1093/nar/gkz469

**Published:** 2019-05-25

**Authors:** Irving Donadon, Erica Bussani, Federico Riccardi, Danilo Licastro, Giulia Romano, Giulia Pianigiani, Mirko Pinotti, Pavlina Konstantinova, Melvin Evers, Shuo Lin, Markus A Rüegg, Franco Pagani

**Affiliations:** 1 Human Molecular Genetics, International Centre for Genetic Engineering and Biotechnology, Padriciano 99, 34149 Trieste, Italy; 2 CBM S.c.r.l., Area Science Park, 34149 Basovizza, Trieste, Italy; 3 Department of Life Sciences and Biotechnology, University of Ferrara, 44121 Ferrara, Italy; 4 Department of Research & Development, uniQure biopharma B.V., Amsterdam, The Netherlands; 5 Biozentrum, University of Basel, Klingelbergstrasse 70, 4056 Basel, Switzerland

## Abstract

Spinal Muscular Atrophy results from loss-of-function mutations in *SMN1* but correcting aberrant splicing of *SMN2* offers hope of a cure. However, current splice therapy requires repeated infusions and is expensive. We previously rescued SMA mice by promoting the inclusion of a defective exon in *SMN2* with germline expression of Exon-Specific U1 snRNAs (ExspeU1). Here we tested viral delivery of SMN2 ExspeU1s encoded by adeno-associated virus AAV9. Strikingly the virus increased *SMN2* exon 7 inclusion and SMN protein levels and rescued the phenotype of mild and severe SMA mice. In the severe mouse, the treatment improved the neuromuscular function and increased the life span from 10 to 219 days. ExspeU1 expression persisted for 1 month and was effective at around one five-hundredth of the concentration of the endogenous U1snRNA. RNA-seq analysis revealed our potential drug rescues aberrant SMA expression and splicing profiles, which are mostly related to DNA damage, cell-cycle control and acute phase response. Vastly overexpressing ExspeU1 more than 100-fold above the therapeutic level in human cells did not significantly alter global gene expression or splicing. These results indicate that AAV-mediated delivery of a modified U1snRNP particle may be a novel therapeutic option against SMA.

## INTRODUCTION

Spinal muscular atrophy (OMIM #253300, #253550 and #253400) is an inherited neuromuscular disease characterized by progressive muscle weakness and muscle atrophy resulting from degeneration of lower spinal cord motor neurons. SMA is the primary genetic cause of infantile mortality, affecting ∼1 in 6000–10 000 new-borns with a carrier frequency of 1:40–1:60 (1). The disease is caused by homozygous deletion or mutation of survival motor neuron 1 (*SMN1*) gene, which codes for the Survival of Motor Neuron (SMN) protein. The nearly identical survival motor neuron 2 gene (*SMN2*), located on the same 5q13 chromosome, differs most importantly from *SMN1* by a synonymous C to T substitution in exon 7 (c.840C>T). This *SMN2* mutation affects an exonic splicing regulatory element and induces ∼80% skipping of exon 7, resulting in a low production of functional SMN protein (2). Clinically, SMA is divided into different subtypes on the basis of severity and age of onset and variation in the copy number of *SMN2* determine most of the phenotypic variability in patients (3) and in mouse models (4). The motor neuron is the critical cell that degenerates in SMA and the principal clinical manifestations of SMA likely result from diminished SMN protein within motor neurons themselves. Analysis of mouse models provided support for the notion that motor neurons are particularly vulnerable to low SMN levels (4–6). However, several studies suggest that the SMN deficiency also affects peripheral tissues. Mice with four SMN2 copies (here defined as ‘mild’ SMA mice) have a mouse-specific phenotype with tail loss due to angiogenesis-vascularization defects (4) and cardiac defects have been also reported in human patients (7,8). Severe SMA animal models have also liver (9–11), cardiac (12), muscle (13) and enteric (11,14) dysfunctions. Splicing rescue in these peripheral tissues is effective and sufficient to compensate the lack of a central nervous system rescue (15,16).

SMN, due to its interaction with several cellular proteins, has been implicated in various processes, including ribonucleoprotein (RNP) formation, cytoskeleton dynamics, ubiquitin homeostasis and endocytosis (17). Depletion of SMN results in complex transcriptome alterations that include defects in splicing (18–23), activation of DNA damage response and cell-cycle arrest (12,22,24–26), and acute phase inflammation (11). Although a comprehensive mechanistic explanation for the transcriptome derangements of SMN deficiency is still lacking, an effective therapeutic strategy has to counteract or mitigate the most relevant RNA alterations in SMA.

Therapeutic strategies for SMA include delivery of the SMN1 coding sequence through a self-complementary adeno-associated serotype 9 (scAAV9) virus (27,28) and activation of SMN exon 7 by chemicals (29,30) or antisense oligonucleotides. Indeed, the current therapy for SMA is based on SMN2 exon 7 splicing correction mediated by an antisense oligonucleotide (Spinraza™) that binds to an intronic splicing silencer (31). This approach, originally tested in SMA mild (32) and severe (15) mice, was approved in December 2016 by the Food and Drug Administration (FDA). The repeated intratechal injections of the oligonucleotide (six for the first year and three for the following years) represent a problem that could be avoided by long-lasting splicing correction approaches with adeno-associated virus (AAV) vectors. Indeed, AAV vectors are a safe and reliable way to durably deliver expression of therapeutic proteins or RNAs (33,34). Here we postulated that a single intratechal injection of an AAV coding for a splice-switching RNA would give a pronounced and sustained effect on SMN2 splicing.

We previously developed an approach based on engineering the U1 core spliceosomal particle to correct exon skipping. The modified particles, named Exon-Specific U1 snRNAs (ExspeU1), bind to intronic sequences downstream of the 5′ss of defective exons promoting their inclusion in the final transcript (35). Unlike the antisense oligonucleotide, ExspeU1s do not block the intronic element but promote spliceosomal activation, recruiting splicing factors that improve the defective definition of the exon (36,37). ExspeU1s are expressed from an ∼600 bp-long cassette gene and code for a short RNA of 164 bp that is assembled into deliberately modified U1 snRNP-like particles. ExspeU1 specificity is obtained by engineering its 9–20 bp 5′ end tail to bind by complementary to the target intronic sequences (37).

Previous *in vitro* and *in vivo* studies indicate that ExspeU1s correct exon skipping in Cystic Fibrosis (35), Haemophilia B (36), Familial Dysautonomia (38) and Netherton Syndrome (39). We have also used ExspeU1s to correct splicing to alleviate SMA (36,37,40). A unique germline copy of an ExspeU1 (ExspeU1sm25—named here ExspeU1sma) efficiently increased SMN2 exon 7 inclusion, SMN protein production and life span in a severe SMA mouse. ExspeU1sma binds in SMN2 intron 7 at nucleotides 25–42 (Supplementary Figure S1) and its overexpression from a single gene in mice had only a minimal effect on the murine transcriptome, providing the first evidence of a safety profile of the molecule (37).

Here, we tested the efficacy and safety of ExspeU1sma delivered by adenovirus associated AAV9 particles in two SMA animal models. AAV9-ExspeU1sma rescued the SMA phenotype in both mild and severe mice, reverting some of the most relevant transcriptome changes caused by SMN deficiency. Furthermore, we find that ExspeU1sma expression can be overdosed several 100-fold without damaging effects in a human cellular model.

## MATERIALS AND METHODS

### Animal models

All animals were housed and handled in a controlled environment in conformity with ICGEB institutional guidelines and in compliance with national and international laws and policies (EU Directive 2010/63/EU) upon approval by the Italian Ministry of Health. The mild SMA model (smn^−/−^; SMN2^2TG/2TG^) was purchased from Jackson's Laboratory (Stock Number: 005058). The severe Taiwanese-SMA model (smn^−/−^; SMN2^2TG/0^) was obtained with multiple crossings as described (4). Newborn mice were intraperitoneally injected at post-natal day 0 and 2 (PND0 and PND2). Injections with a final volume of 25 μl were performed with a micro-syringe (Hamilton) with a 33-gauge removable needle. Mice were sacrificed to collect tissues and organs (brain, liver, heart, kidney, muscle and spinal cord).

### AAV production

AAV9 vector encoding ExspeU1sma and wild-type U1 plasmid plasmids were packaged into AAV9 by a baculovirus-based AAV production system (uniQure, Amsterdam, The Netherlands) as previously described (41). Briefly, to generate AAV9 particles, insect cells were infected with baculoviruses encoding rep for replication and packaging and cap-9 for the AAV9 capsid and the expression cassette. After viral particle production, preparations were purified with AVB Sepharose high-performance affinity medium using AKTA Explorer purification system (both, GE Healthcare Piscataway, NJ, USA). Titers of AAV vector stocks were determined by quantitative polymerase chain reaction (qPCR) using specific-primers U1WT FW2 and U1WT RV2 (Supplementary Table S1).

### DNA isolation and AAV quantification

DNA was isolated from tissues according to the DNeasy Blood and Tissue kit protocol (Qiagen, 69506). The DNA concentration was analyzed with a Nanodrop 2000 (Thermo Scientific). To determine the vector distribution in all samples, a SYBR Green (Applied Biosystems, 4385612) qPCR was performed, using the 7500 Fast Real/Time PCR System (Thermo Scientific using the 7500 Fast Real/Time PCR System (Thermo Scientific) with ‘U1WT ITR FW’ and ‘U1WT U1 ter RV’ primers. 50 × 10^3^ pg of sample DNA was used per reaction and qPCR was run with one step at 95°C for 3 min and 40 cycles of 95°C for 3 s and 60°C for 30 s. The Viral Copy Number (VCN)/μg DNA was quantified with plasmid standard line (7 reactions, 10^8^ to 10^2^ genomic copies per reaction) and the VCN per cell (VCN/cell) calculated considering the estimated quantity of DNA in the nucleus of a murine cell (∼6.1 pg DNA/diploid cell) (42).

### Creation of ExspeU1sma HEK293 Flp-In T-REx stable clones

HEK293 Flp-In T-REx cells were grown according to manufacturer's instructions (ThermoFisher). For stable integration, we cloned three copies of the ExspeU1sma into the pcDNA5/FRT/TO vector (ThermoFisher) using different restriction sites (BamHI, HindIII-KpnI and XhoI-ApaI). The included 3XExspeU1sma cassette was verified by sequencing. Stable clones were produced by co-transfecting the 3XExspeU1sma plasmid in HEK293 Flp-In T-REx cells with the pOG44 plasmid, whereas control empty clones were obtained by co-transfecting the pOG44 plasmid with an empty pcDNA5/FRT/TO vector. After 48 h transfected cells were selected with Blasticidin. The stable clones were analyzed after 3–5 passages (split 1:4). Transient transfection of stable clones with the pCI-SMN2 minigene was performed as previously described (35).

### RNA isolation and mRNA analysis

Total RNA extraction was performed using TRIzol reagent (Thermofisher) according to manufacturer's instructions. Reverse-transcription was performed using 500 ng of total RNA with the Superscript Vilo MasterMix (Thermofisher). For specific detection of ExspeU1sma, total RNA was treated with DNase and quantitative PCR was performed with iQ-SYBR Green Supermix (Bio-Rad Laboratories, Inc., Hercules, CA, USA) using specific primers (Supplementary Table S1). The expression levels of ExspeU1sma were calculated as previously described (40). Quantitative PCR conditions were: 1 cycle at 98°C for 3 min; 40 cycles at 95°C for 5 s and at 60°C for 25 s. Endogenous GAPDH was used as housekeeping gene. pCI-SMN2 minigene splicing analysis and endpoint PCR on *in vitro* endogenous SMN transcripts were carried out as previously described (35,37); *in vivo* end-point analysis of SMN2 transcripts was performed as described by (43). Quantitative analysis of total and full-length (FL) SMN2 transcripts in animal samples was performed according to (44).

### Protein isolation and western blot analysis

Protein samples were obtained by tissue lysis with ice-cold RIPA buffer (Sigma, St Louis, MO USA) and Proteinase Inhibitor Cocktail (Roche). A total of 20 μg of protein was separated on NuPAGE 4–12% Bis-Tris precast gels (ThermoFisher, Waltham, MA USA) and transferred to nitrocellulose membranes. The membranes were blocked for 1 h in 5% non-fat milk and successively blotted overnight at 4°C in 5% milk with a primary SMN-specific antibody (ab610646,BD Biosciences, USA, 1:2000) and a primary GAPDH antibody (ab8245, Abcam, USA, 1:5000).

Membranes were washed and incubated with specific secondary antibodies for 1 h at room temperature (anti-Mouse 1:5000). Protein bands were detected with ECL Western blotting substrate (ThermoFisher, Waltham, MA, USA) followed by exposure to X-ray film (Kodak) or to UVItec CAMBRIDGE Alliance.

### Immunohistochemistry and neuromuscular junction analysis

Lumbar spinal cord segments (L3-5) of P36 animals were dissected, fixed in 4% Paraformaldehyde (PFA), cryopreserved in 30% sucrose and processed for 20 μm cryosections. After antigen retrieval with pH 6.0 citrate buffer, each section was incubated with rabbit anti-ChAT antibody (1:300; Synaptic Systems). Immunoreactivity was detected by goat anti-rabbit IgG (H+L) highly cross-adsorbed secondary antibody, Alexa Fluor 488 (1:500; Thermo Fisher Scientific). For each spinal cord we counted 4–6 sections that were 100 μm apart, to avoid double counting of the same neurons.

Dissection on P36 animals was performed on longissimus capitis and tibialis anterioris muscles. Samples were incubated with alpha-bungarotoxin for 30 min at room temperature followed by phosphate-buffered saline (PBS) washing, fixed with PFA for 10 min, rinsed with PBS and stored at 4°C. Muscles were cut into bundles, soaked in 5 ml of 100 mM glycine (Sigma, G7126) in PBS with gentle shaking for 1 h in the cold room. Muscle bundles were incubated with 5 ml of blocking solution (3% bovine serum albumin (Sigma, A9647), 0.02% triton X-100, 0.01% NaN_3_ in PBS) with gentle shaking for 3 h in the cold room. Muscle bundles were incubated with 0.5 ml of primary antibodies in blocking solution (Neurofilament, Sigma N4142, 1:8000 and Synptophysin, Genetex GTX100865, 1:200) with gentle shaking for 60 h in the cold room. Muscle bundles were washed with 5 ml of blocking solution with gentle shaking, five times for 2 h in the cold room, and then incubated with 0.5 ml of secondary antibodies in blocking solution containing Alexa conjugate goat anti-rabbit IgG, molecular probes A-11034, 1:1000; DAPI, 1:4000 with gentle shaking for 60 h in the cold room. The muscle bundles were then washed with 5 ml of blocking solution with gentle shaking, 5 × 2 h in the cold room. To analyze the neuromuscular junction morphology quantitatively, muscle bundles were observed under a Leica DM5000b fluorescent microscope with a 100× objective. Each junction was classified according to the parameters indicated in the results.

### Neuro functional tests

The four-limb hanging test was performed between the fifth and the ninth week after birth. The time recorded is the ability of mice to hold themselves on to the bars after the metal grid was inverted, with 1 min being the maximum time. The Rotarod test was performed between the fifth and the ninth week after birth. The ability to maintain balance on a rotating cylinder was measured with an accelerating rotarod apparatus manufactured indigenously at ICGEB, Trieste, Italy. The cylinder was 4.0 cm in diameter and covered with sand paper to improve the grip of the animals. Mice were confined to a section of the cylinder 7.5 cm long by plexiglass dividers and placed on the rod at a speed of 4 RPM which accelerates over the course of 300 s to 40 RPM (e.g. an increase of 1 RPM is achieved approximately every 8.3 s). Using an acceleration profile, three trails per day were given and each trial ended when the mouse fell off the rod, and latency was recorded, with a maximum time of 300 s. In all tests mice were tested three times per session and the results were averaged.

### RNA-sequencing and data analysis

Messenger RNA sequencing (mRNA-seq) was performed by IGA Technology Services (Udine, Italy) or Macrogen. HEK293 Flp-In T-REx stable clones expressing ExspeU1sma and severe SMA mice tissues were purified with TRIazol (Ambion) and quality of total RNA was assessed using Agilent 2100 nano bioanalyzer microfluidic chips and a Nanodrop UV spectrophotometer (ThermoFisher). Only RNA with a RIN value of 8.0 or higher and a 28s/18s ratio ∼1.8 was taken forward for sample preparation. The size distribution of HEK293 cells and mouse-derived libraries were estimated by electrophoresis on an Agilent high sensitivity bioanalyzer microfluidic chips and yield was quantified using the KAPA library quantification kit (KK4824, Kapa Biosystems, Boston, MA, USA). The libraries were pooled at equimolar concentrations and diluted before loading onto the flow cell of the HiSeq 2500 (Illumina) for both clustering and sequencing. Amplified clusters in the flow cell were then sequenced with 125-base paired-end reads using the TruSeq Rapid SBS Kit—HS (Illumina Inc.). Real-time image analysis and base calling were performed on a HiSeq 2500 instrument using the recommended sequencing control software. Illumina standard software was used for de-multiplexing and production of FASTQ sequence files. FASTQ raw sequence files were subsequently quality checked with FASTQC software (version 0.11.3 http://www.bioinformatics.bbsrc.ac.uk/projects/fastqc). Subsequently, sequences including adaptor dimers, mitochondrial or ribosomal sequences were discarded. The resulting set of trimmed reads from HEK293 cells and mouse tissues were then mapped onto *Human* GRCh38/hg38 and *Mus musculus* GRCm38 (mm10), respectively, using Spliced Transcripts Alignment to a Reference(STAR) algorithm (45), which we use due to its accuracy in aligning reads between 50 and 100 bases to long genomes. Differential gene expression analysis from HEK293 cells and mouse tissues was performed using the Bioconductor package DESeq2 (version 1.4.5) using default parameters (46). To detect outlier data after normalization we used R packages and before testing differential gene expression we dropped all genes with low normalized mean counts to improve testing power while maintaining type I error rates. Estimated false discovery rate (FDR) values for each gene were adjusted using the Benjamini–Hochberg method. Prior to analysis, genes without a poly-A tail were discarded. Features with baseMean greater than 100, padj value under 0.01 and absolute logarithmic base 2 Fold Change (log_2_FC) ≤−2 or ≥2 were considered having a significant altered expression. For genomewide splicing analysis, BAM files produced from STAR mapping were input into rMATS (47), using *Human* GRCh38/hg38 annotation for HEK293 Flp-In T-REx stable clones or *M*. *musculus* GRCm38 (mm10) for mouse tissues. For detection of alternative splicing (AS) patterns, human or mouse annotations were generated containing all consecutive spliced and unspliced exon-intron-exon triads from hg38 (Gencode v29) and mm10 (Gencode v19). FIve basic types of AS were analyzed: skipped exons (SE), retained introns (RI), mutually exclusive exons (MXE), alternative 5′ (A5SS) and alternative 3′ splice sites (A3SS). For HEK293 analysis, due to the very high similarity between SMN1 and SMN2 transcripts, the number of counts of SMN1 and SMN2 was combined since the software is unable to differentiate the alternative splicing pattern of exon 7 derived from these two genes. Read coverage was based on actual reads as used in (48): SE, RI and MXE types with an actual reads mapping to all exclusion splice junction ≥20 were considered, whereas for A5SS and A3SS types, ≥40 actual reads mapping to the sum of all splice junctions involved in the specific event were considered. Estimated FDR values for each gene were adjusted using the Benjamini–Hochberg method. The threshold parameters were set at FDR value under 0.05 and absolute Inclusion Level Difference (Δψ) ≤−2 or ≥2.

### Pathway and network analysis by Ingenuity Pathway Analysis

The list of significant differentially expressed genes in muscle and liver of heterozygous, SMA severe and SMA severe-treated mice, containing gene identifiers and corresponding expression values, was uploaded into the IPA software (Qiagen). The ‘core analysis’ function included in the software was used to interpret the differentially expressed data, which included biological processes, canonical pathways and gene networks. Each gene identifier was mapped to its corresponding gene object in the Ingenuity Pathway Knowledge Base (IPKB). Comparison analysis function included in the software was used to produce heat map and evaluate activation *Z*-score and -log(*P*-value) of canonical pathways.

### Statistical analysis

The statistical analysis was performed with Prism (GraphPad, USA) version 7.0. The exact sample size and/or biological replicates are reported in figure legends. The comparisons of two groups were performed using Student’s *t*-test, whereas Analyses of Variance (ANOVA) was used to compare three or more groups, applying Bonferroni correction. A standard confidence interval of 95% was used for all statistical analysis and the significance *P*-value were indicated by asterisks (*P < 0.05; **P < 0.01; ***P < 0.001). Data of qPCR (Figures 3B–D and 9E) were normalized and compared to saline treated mice for each tissue and to control clones, respectively.

## RESULTS

### Identification of ExspeU1sma RNA therapeutic levels in transgenic mice

**Figure 1.**
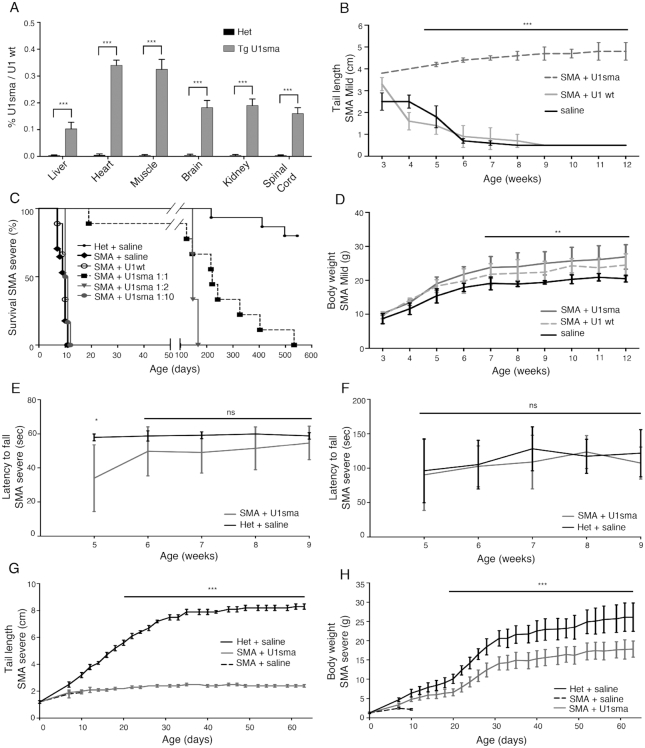
Systemic AAV9-ExspeU1sma treatment rescues SMA mice. (**A**) Identification of ExspeU1sma therapeutic levels in SMA rescued transgenic mice carrying one copy of the ExspeU1sma (Tg U1sma) (smn^−/−^, SMN2^+/+^, ExspeU1sma^+/-^). Data (mean ± SD) are expressed as percentage of ExspeU1sma relative to endogenous U1. For each tissue, statistical analysis, performed using Student’s *t*-test, compares TgU1sma mice (*n* = 6) to heterozygous mice (smn^−/+^, SMN2^+/+^, ExspeU1sma^−/−^) (*n* = 6) (P40). (**B**) Rescue of tail length in SMA mild (smn^−/−^; SMN2^2TG/2TG^) treated with AAV9-ExspeU1sma (*n* = 10), AAV9-U1wt (*n* = 6) and saline (*n* = 8) with two intraperitoneal injections (P0 and P2) at 1.5 × 10^12^ vgp/mouse/injection. Statistical analysis was performed by One Way ANOVA with Bonferroni correction. (**C**) Kaplan–Meier survival curves. Severe SMA mice (smn^−/−^, SMN2^2TG/0^) were treated with two intraperitoneal injections (P0 and P2) of AAV9-U1wt at 1.5 × 10^12^ (U1wt, *n* = 4), and of AAV9-ExspeU1sma at 1.5 × 10^12^ (U1sma 1:1, *n* = 9). Animals were also treated AAV9-ExspeU1sma with a single injection at P0 of 1.5 × 10^12^ vgp/mouse (U1sma 1:2, *n* = 3) and with a double injection (P0 and P2) at 1.5 × 10^11^ (U1sma 1:10, *n* = 6) vgp/mouse/injection. Control heterozygous (smn^+/−^; SMN2^2TG/0^) (*n* = 15) and severe SMA mice (*n* = 17) were treated with saline. Statistical analysis was performed with log rank Mantel–Cox test. ***P < 0.001 for all groups and ***P < 0.001 for severe SMA saline versus AAV9-ExspeU1sma-treated mice. (**D**) Weekly measurements of body weights in SMA mild animals treated with AAV9-ExSpeU1sma (*n* = 9), AAV9-U1wt (*n* = 8) and saline (*n* = 10). Statistical analysis was performed by Two-way ANOVA. (**E**) Hanging test in severe SMA mice treated with AAV9-ExspeU1sma (*n* = 6) and control heterozygotes (smn^−/+^; SMN2^2TG/0^) (*n* = 6). Statistical analysis was performed by multiple *t*-test. (**F**) Neuro-functionality and balance (Rotarod test) severe SMA mice treated with AAV9-ExspeU1sma (*n* = 6) and control heterozygotes (smn^−/+^; SMN2^2TG/0^) (*n* = 6). Statistical analysis was performed by multiple *t*-test. (**G**) Tail length in heterozygous mice (smn^+/−^; SMN2^2TG/0^) (*n* = 11), SMA severe (smn^−/−^; SMN2^2TG/0^) animals treated with AAV9-ExspeU1sma (*n* = 9) and SMA saline (*n* = 10). Statistical analysis was performed by Student *t*-test. (**H**) Weekly measurements of body weights of heterozygous mice (smn^+/−^; SMN2^2TG/0^) (*n* = 9), SMA severe animals treated with saline (*n* = 9) and treated with AAV9-ExSpeU1sma (*n* = 7). Statistical analysis was performed by Two-way ANOVA. Values reported in panels (B), (D), (E), (F), (G) and (H) are means ± SD. (***P < 0.001; **P < 0.01; ns, not significant).

We initially wanted to understand what dose of ExspeU1sma RNA is required for SMN2 splicing correction and phenotypic improvement. For this, we analyzed our previously reported severe SMA transgenic animals that have one *ExspeU1sma* and two *SMN2* genes. In this model, germline expression of the ExspeU1sma induces long lasting survival with significant and ubiquitous correction of SMN2 splicing and rescue of the phenotype (37). As ExspeU1 particles and normal U1snRNP are structurally similar, we first quantified ExspeU1 RNA relative to endogenous U1wt RNA by qRT-PCR. The transgenic animal expressed a very low percentage of ExspeU1sma compared to endogenous U1 in various tissues (0.1–0.3%/U1wt) (Figure 1A). Given the significant phenotypic improvement present in this transgenic animal, we consider that the tissue therapeutic levels of ExspeU1sma RNA that have to be obtained by an AAV-mediated delivery need not be much >0.1–0.3% of the wild-type level. We refer to this hereon as the therapeutic level.

### Systemic delivery of AAV9-ExspeU1sma rescues SMA mice

To evaluate the efficacy viral delivery of ExspeU1sma, we considered two SMA mouse models. The mild SMA mouse (smn ^-/-;^ SMN2^2TG/2TG^) carries four *SMN2* copies and the only evident phenotype is tail necrosis that results in a complete loss of the tail by the 6th week after birth (4). The severe Taiwanese SMA mice (smn^−/−^, SMN2^2TG/0^) on the other hand does not survive beyond 10 days. These two models have been extensively used to establish the therapeutic profile of antisense oligonucleotide splice-switching therapies leading to the development of the FDA-approved Spinraza™ (15,16,44). New-born mice were injected intraperitoneally at birth, and 2 days after, with AAV9-ExspeU1sma, AAV9-U1wt, both at 1.5 × 10^12^vgp/mouse or saline. Treatment with AAV9-ExspeU1sma, completely recovered the tail length of the mild SMA mouse and significantly improved the survival of the severe SMA (Figure 1B and C). In the mild model, 6 weeks after birth, AAV9-U1wt or saline-treated animals completely lose the tail, whereas the ExspeU1sma-treated animals maintained a 4–5 cm long tail with no necrosis over the following months. AAV9 treatments also slightly improved body weight in this model (Figure 1D). In the severe Taiwanese SMA mice, where untreated animals or animals treated with AAV9-U1wt survived only around 10 days, ExspeU1sma treatment lengthened survival to a median of 219 days (22-fold increase, Figure 1C). The reduction of the dose to 50% in a single administration at P0, resulted in 150 days of survival, whereas a 10% of the original dose in two injections was ineffective. The rescued mice had normal neuromuscular functions like heterozygous (smn^+/−^, SMN2^2TG/0^) animals (Figure 1E and F). In addition, analysis of neuromuscular junction and the motor neuron count, that are affected in the severe Taiwanese mouse model (16,44), revealed no difference between the adult heterozygotes and corresponding treated SMA animals (Supplementary Figure S2). Treated tails of Taiwanese animals were about the quarter of the length and weighed around 40% less than age-matched heterozygotes (Figure 1G and H). Overall these data clearly demonstrate that systemic intraperitoneal injection of viral ExspeU1sma particles rescues the SMA phenotypes of both mild and severe SMA mice.

### AAV9-ExspeU1sma bio-distribution, expression and SMN2 splicing rescue

**Figure 2.**
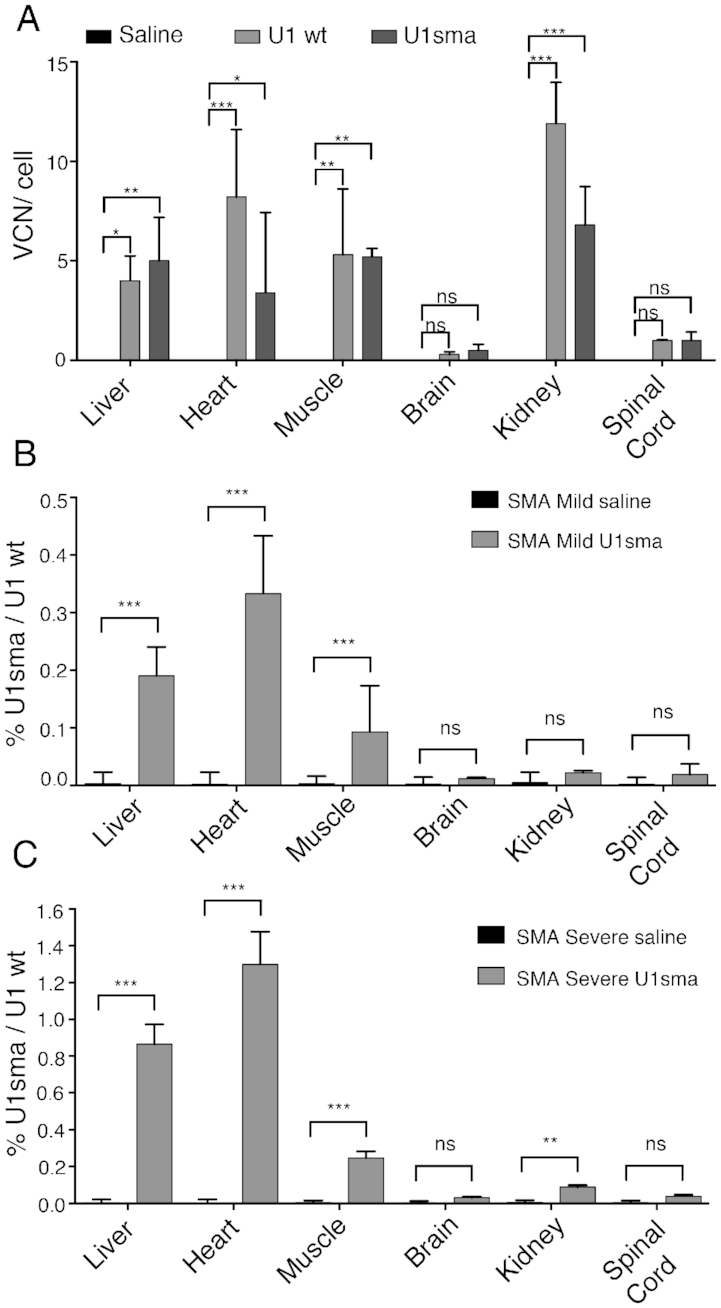
Tissue distribution of AAV9 DNA and ExspeU1sma RNA expression. (**A**) SMA mild mice (smn^−/−^; SMN2^2TG/2TG^) were treated with AAV9-ExspeU1sma (*n* = 3), AAV9-U1wt (*n* = 3) and saline (*n* = 2) with two intraperitoneal injections (P0 and P2) at 1.5 × 10^12^ vgp/mouse/injection. Animals were sacrificed and organs collected at P7. Data are expressed as mean ± SD of VCN per cell. Statistical analysis was performed using Two-Way ANOVA. Level of ExspeU1sma expression in (**B**) SMA mild (smn^−/−^; SMN2^2TG/2TG^) and (**C**) SMA severe (smn^−/−^; SMN2^2TG/0^) mice treated with saline and with AAV9-ExSpeU1sma (two intraperitoneal injections at P0 and P2, 1.5 × 10^12^ vgp/mouse/injection). Data (mean ± SD) are expressed as percentage of ExSpeU1sma relative to endogenous U1. For each tissue statistical analysis, performed using Student’s *t*-test compares saline to U1sma-treated mice at P7 (*n* = 3 per genotype) (***P < 0.001; **P < 0.01; *P < 0.05; ns, not significant).

To clarify the tissue specific effect of the AAV9 treatment along with the therapeutic level obtained we comprehensively evaluated the virus's distribution, the level of ExspeU1sma RNA and the SMN splicing and protein improvement. To study the viral location, we measured the AAV9 DNA by qPCR in different tissues from 7-day-old-treated SMA mild mice (Figure 2A). Most of AAV9 DNA was present in liver, heart, muscle and kidney (∼4–11 copies of AAV9 per cell). In contrast, brain and spinal cord had negligible VCN. We then evaluated the amount of ExspeU1sma expressed in different tissues. In agreement with the AAV9 bio-distribution, heart, liver and muscle had the highest ExspeU1sma RNA concentrations (Figure 2B). In contrast, brain, kidney and spinal cord had small amounts of ExspeU1sma RNA. Similar expression levels were observed in the same tissues of the severe SMA mice (Figure 2C). In liver, heart and muscle of both SMA mice models, ExspeU1sma was expressed above the therapeutic levels (0.1–1.2% of ExspeU1sma/U1wt).

**Figure 3.**
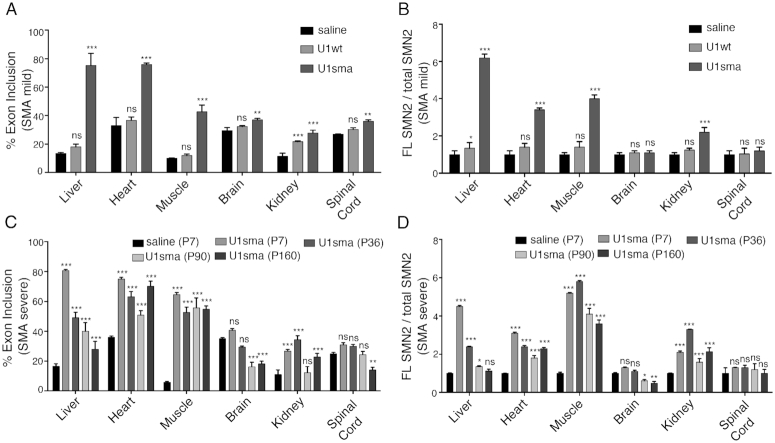
Tissue specific SMN2 splicing rescue in SMA mouse models. (**A**) Endpoint PCR of SMN2 splicing isoforms in SMA mild animals treated with saline, AAV9-U1wt and AAV9-ExSpeU1sma at P7. Data era expressed as percentage of exon 7 inclusion. (**B**) qPCR quantification of the ratio of SMN2 full length (FL) transcripts to total SMN2 transcripts in SMA mild animals treated with saline, AAV9-U1wt and AAV9-ExSpeU1sma at P7. Expression level of saline-treated animals is set to 1. (**C**) Endpoint PCR of SMN2 splicing isoforms in SMA severe animals treated with saline and mice treated with AAV9-ExSpeu1sma at the indicated time. Data are expressed as percentage of exon 7 inclusion. (**D**) qPCR quantification of the ratio of SMN2 full length (FL) transcripts to total SMN2 transcripts in SMA severe animals treated with saline and with AAV9-ExSpeu1sma at the indicated time. Expression level of saline-treated animals is set to 1. Data are expressed as mean ± SD of three technical replicates on 3 mice and statistical analysis performed by Two-Way ANOVA. The asterisks indicate comparison to the saline treated SMA mice (***P < 0.001; **P < 0.01; *P < 0.05; ns, not significant).

Next, we analyzed the effect of the ExspeU1sma on splicing of SMN2 by end-point RT-PCR in the mild SMA mice and found at P7 increased inclusion of Exon 7 mainly in liver, heart and muscle (Figure 3A and Supplementary Figure S3). Consistent with the distribution of the virus, its ExspeU1sma expression and its splicing outcome, we observed a 2- to 6-fold increase in full-length SMN2 RNA in liver, heart, muscle and kidney, in treated P7 animals (Figure 3B). As expected, the increase was smaller in spinal cord, with no change in brain. The control virus encoding the U1 wild-type did not change SMN2 splicing or full-length SMN2 RNA levels (Figure 3A and B; Supplementary Figures S3 and 4) which excludes that the changes in SMN2 splicing are due to a non-specific effect of either the U1 snRNP particle or the viral particles.

Next, we analyzed the long-lasting effect of the ExspeU1sma on SMN2 splicing and full-length SMN2 RNA in the severe SMA mice (Figure 3C and D). Importantly, SMN2 splicing and full-length SMN2 RNA persisted for over 5 month after treatment in the post-mitotic heart, muscle and kidney (Figure 3C and D, P36, P90 and P160 bars and Supplementary Figure S4A). The effect was less durable in liver (Figure 3C and D, P36, P90 and P160 bars and Supplementary Figure S4A). This event on liver is consistent with a progressive age-dependent dilution of episomal AAV in replicating tissues (49).

**Figure 4.**
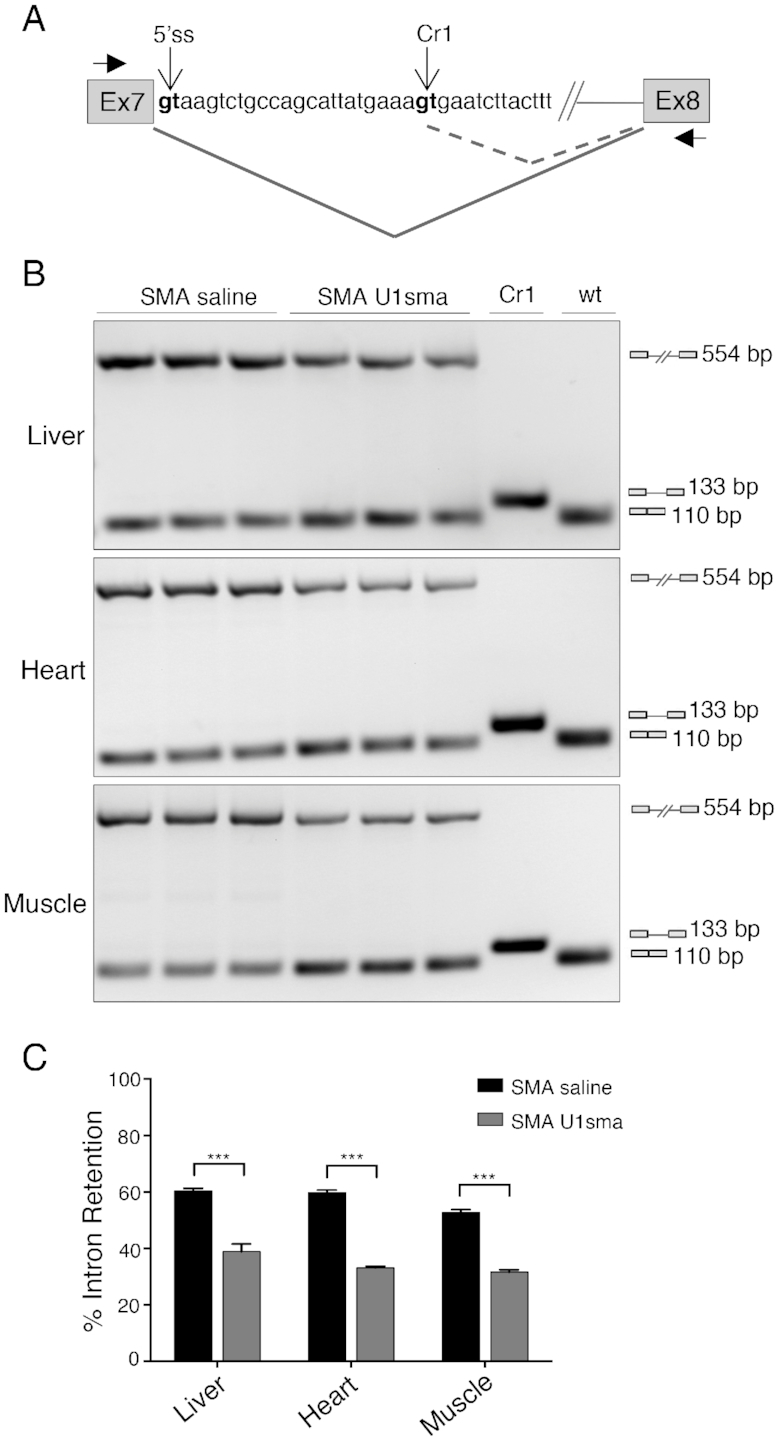
AAV9-ExSpeU1sma *in vivo* does not induce mis-splicing of SMN2 intron 7. (**A**) Schematic representation of SMN2 exon 7-intron 7-exon 8 structure. The wild-type (5′ss) and cryptic donor sites identified in (50) (Cr1), as well as the position of the primers (Ex7 and Ex8) used in the amplification experiments are indicated. Differential splicing events are shown. (**B**) SMA severe mice (smn^−/−^, SMN2^2TG/0^) treated with saline (*n* = 3; P7) and AAV9-ExSpeU1sma (*n* = 3; P7) were analyzed by endpoint PCR with Ex7 and Ex8 primers. DNase-treated RNA samples correspond to samples analyzed in Figure 3C (P7 bars) and Supplementary Figure S4A (P7 lanes). Amplification experiments in SMA saline and SMA U1sma samples showed an upper (554 bp) and a lower (110 bp) band that corresponds to retained intron 7 and correctly spliced transcripts, respectively. Cr1 and wt lanes are control amplifications of plasmids that contain the spliced cryptic (133bp) or the normal 5′ss (110 bp) sequences, respectively. Amplification experiments of AAV9-ExSpeU1sma-treated samples did not show any Cr1 133bp-long band. (**C**) Histogram shows quantification of the percentage of intron 7 retention. Data are expressed as mean ± SD of two technical replicates on three mice and statistical analysis was performed using Student’s *t*-test (***P < 0.001).

ExspeU1s are known to promote exon and intron definition (37) and the end point PCRs did not show any evident additional band (Supplementary Figures S3 and 4) generated by activation of cryptic splice sites. However, in minigene experiments some ExspeU1s might activate a cryptic 5′ss located 23 nts downstream the wt-5′ss and eventually stimulate intron 7 retention (50). To exclude potential mis-splicing events induced *in vivo* by AAV9-ExspeU1sma, we performed end point PCR experiments with specific primers on RNA samples derived from those SMA mouse tissues with the most effective SMN2 exon 7 splicing improvement (Figure 4A). Amplification experiments in liver, heart and muscle clearly showed no cryptic 5′ss splice site activation (Figure 4B). In addition, AAV9-ExspeU1sma treatment improved intron 7 splicing, consistent with its previously reported positive effect on intron definition (37) (Figure 4C).

**Figure 5.**
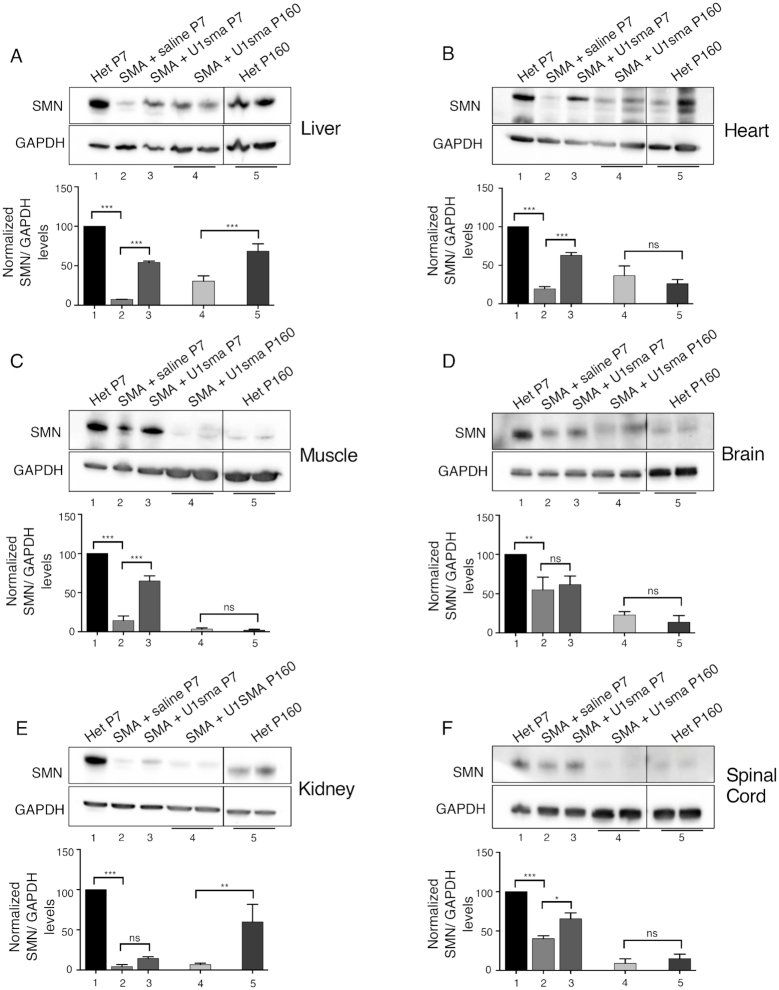
AAV9-ExspeU1sma rescues SMN protein in severe SMA mice. The indicated mice were treated with two intraperitoneal injections of AAV9-ExspeU1sma (P0 and P2) at 1.5 × 10^12^ vgp/mouse/injection and tissues samples analyzed at P7 and P160 (Panels **A**-**F**). For each tissue, upper part shows representative western blot analysis of SMN protein and lower part graph quantification of band intensities. Lane 1: P7 heterozygous mice (smn^+/−^; SMN2^2TG/0)^) (*n* = 2), lane 2: P7 SMA mice (smn^−/−^; SMN2^2TG/0)^) (*n* = 3), lane 3: P7 U1sma-treated SMA mice (*n* = 3), lane 4: P160 U1sma-treated SMA mice (*n* = 2), lane 5: P160 heterozygote mice (*n* = 2). Dividing lines indicate cropping and annealing of the same western blots’ images acquired with UVITEC CAMBRIDGE Alliance software. Expression level of heterozygotes at P7 is set to 100. GAPDH was used as internal reference control. Additional western blots used for quantitative analysis are shown in Supplementary Figure S5. Data are expressed as mean ± SD. Statistical analysis was performed using One Way ANOVA (***P < 0.001; **P < 0.01; *P < 0.05; ns, not significant).

To establish if the effect observed on splicing in the severe mice affects the SMN protein abundance we performed immunoblotting experiments. As SMN protein levels are known to be developmentally downregulated in a tissue-specific manner (51–53), we evaluate the amount of SMN protein in treated SMA mice relative to corresponding age-matched untreated animals. At P7, compared to affected SMA mice, AAV9-ExspeU1sma treatment induced a significant increase in SMN protein in liver, heart and muscle (Figure 5A–C), a small increase in spinal cord (Figure 5F) and no effect in brain and kidney (Figure 5D and E) (compare lanes 2 and 3). Interestingly, heart, muscle and liver in SMA mice treated with AAV9-ExspeU1sma showed approximately half of the SMN levels found in normal heterozygotes (compare lanes 3 and 1). At P160, the SMA treated mice had in heart, muscle, brain and spinal cord similar SMN proteins levels to age-matched heterozygous animals whereas liver and kidney showed less SMN protein in AAV9-ExspeU1sma-treated animals (∼2- and ∼7-fold less, respectively) (Figure 5). Together these data indicate that systemic viral delivery of ExspeU1sma induces a preferential and significant improvement in SMN2 splicing and SMN protein in the peripheral organs: liver, heart and muscle, and a minor effect on the central nervous system. Crucially this treatment is long lasting in several post-mitotic tissues and rescues the severe SMA phenotype.

### AAV9-ExspeU1sma reverses SMA gene expression and splicing profiles

Next, we evaluated differentially expressed genes, alternative splicing events and deregulated pathways in the severe SMA mice. Differentially expressed genes were analyzed with DESeq2, alternative splicing events with rMATS and deregulated pathways with IPA. We compared SMA affected with normal heterozygous animals and analyzed liver and muscle, two representative tissues where ExspeU1sma treatment reaches the therapeutic level and corrects SMN2 splicing and SMN protein (Figure 3 and 5). Compared to the heterozygous animals, we identified in liver and muscle of SMA mice, respectively 37 and 51 upregulated, and 76 and 26 downregulated genes. Supplementary Data File S29 shows the differentially expressed genes whereas Supplementary Data Files S1 and 2 all gene expression events. Three genes were differentially regulated in both tissues. Prr14l was downregulated in SMA and Gm37795 and a cyclin-dependent kinase inhibitor and mediator of cell-cycle arrest, CdKn1a, were upregulated. Transcriptome-wide splicing changes with the rMATS algorithm showed that most of the SMA splicing changes involved exon skipping (171 and 78 for muscle and liver, respectively) with some intron retention (31 and 46 for muscle and liver, respectively) and mutually exclusive exons (29 and 4 for muscle and liver, respectively). Very few changes were observed for alternative 5′ and 3′ splice site changes (Supplementary Figure S6). Significant alternative splicing events are listed in Supplementary Data File S30 whereas all alternative splicing events are shown in Supplementary Data Files S3–12. To detect the SMA-deregulated pathways, we tested all differentially expressed genes obtained in control versus SMA mice, divided by tissue, using Qiagen's Ingenuity Pathway Analysis (IPA). In muscle, SMA showed a preferential and robust involvement of pathways and processes associated with cell division and DNA damage response (Supplementary Figure S7A). Most pathways were downregulated in SMA, including the mitotic role of polo kinases, nucleotide excision repair pathways, role of BRACA1 in DNA damage response, ATM signaling and cyclins/cell-cycle regulation. In contrast, ‘cell cycle:G2/M DNA damage checkpoint regulation’ and ‘role of CHK proteins in cell cycle checkpoint control’ pathways are up-regulated in SMA. In SMA liver, we observed a preferential activation of inflammation pathways (Supplementary Figure S7B). This included up-regulation of acute phase response signalling, LPS/IL-1-mediated inhibition of RXR function, leukocyte extravasation and Toll-like receptor signaling and production of Nitric Oxide and reactive oxygen species in macrophages.

**Figure 6.**
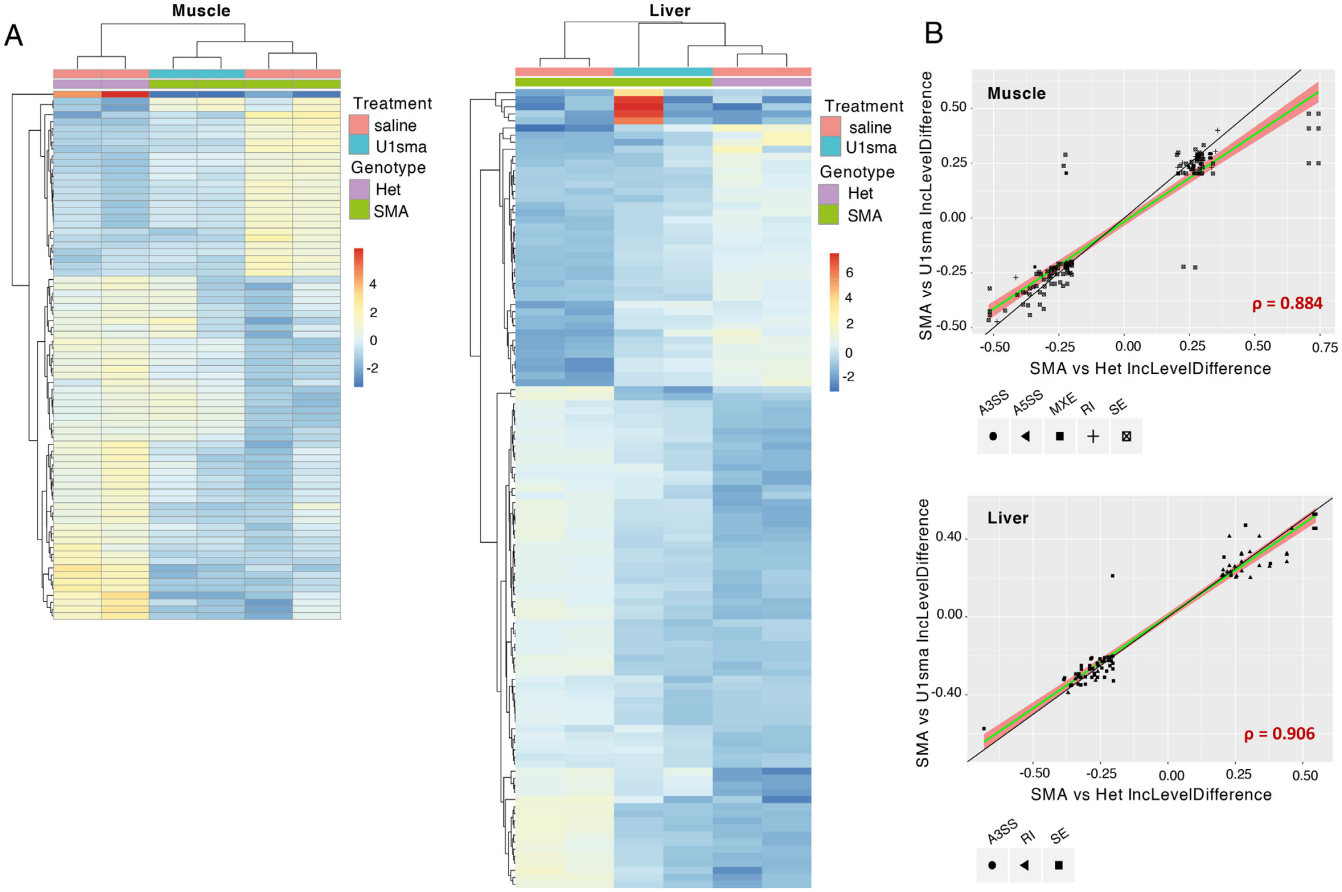
Effect of AAV9-ExspeU1sma on SMA-dependent gene expression and alternative splicing changes in liver and muscle. (**A**) Clustering analysis showing log_2_ fold change of genes that change significantly in mRNA expression in muscle and liver of untreated severe SMA mice, severe SMA mice treated with AAV9-ExspeU1sma (U1sma) and heterozygous control mice. Two 7-day-old mice were tested for each condition. Treatment and Genotype colour schemes are depicted on top right (padj < 0.01; log_2_FC ≤−2 or ≥ 2). (**B**) Scatterplots of inclusion level difference of significant alternative splicing events in muscle and liver of heterozygous mice treated with saline (*n* = 2) and severe SMA mice treated with AAV9-ExspeU1sma (U1sma) (*n* = 2) at P7. Skipped exons (SE), retained introns (RI), mutually exclusive exons (MXE), alternative 5′ (A5SS) and alternative 3′ splice sites (A3SS) are indicated. The black line indicates a completely linear relationship. The green line and the red area indicate, respectively, the linear regression and linear distribution model based on significant splicing events rescued by ExspeU1sma. Spearman's rho correlation for these events is indicated in red.

Next, we evaluated the effect of the AAV9-ExspeU1sma treatment on the SMA-induced transcriptome changes. Clustering analysis of differentially expressed genes showed that most of the SMA-induced changes in gene expression in liver and muscle were reversed or mitigated by the AAV9-ExspeU1sma (Figure 6A). In both tissues, there was also a strong correlation between alternative splicing events that are deregulated in SMA mice and alternative splicing events that respond to AAV9-ExspeU1sma, indicating the AAV9-ExspeU1sma treatment reverses most of the SMA-induced alternative splicing (Figure 6B and Supplementary Data Files S13–22).

**Figure 7.**
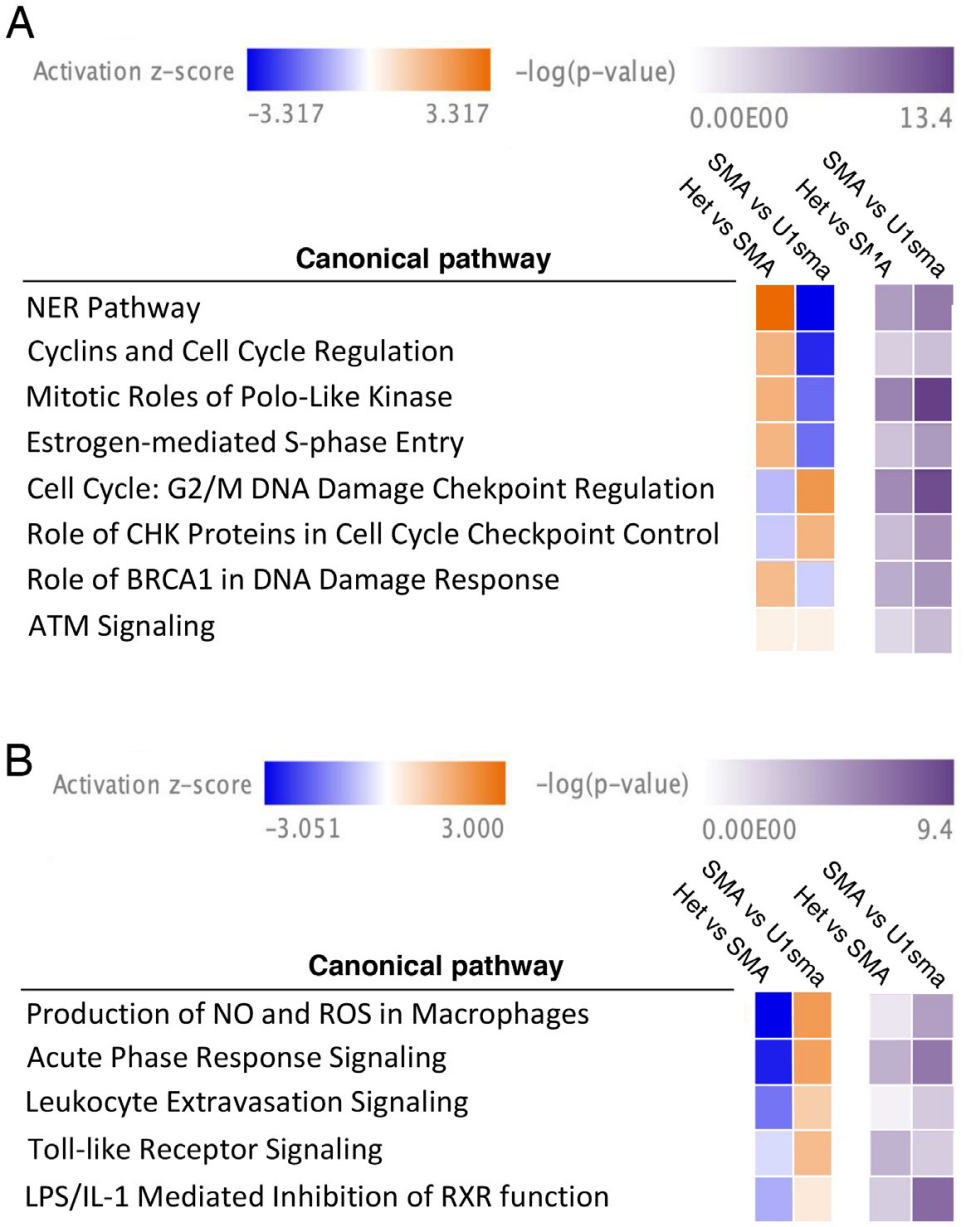
AAV9-ExspeU1sma treatment reverses SMA-deregulated pathways in severe SMA mice. Ingenuity comparison analysis of selected canonical pathways shows a significant rescue of (**A**) pathways associated with cell-cycle division and DNA damage response in muscle and (**B**) pathways associated with inflammation in liver. Activation *z*-score and *P*-value are indicated on top.

**Figure 8.**
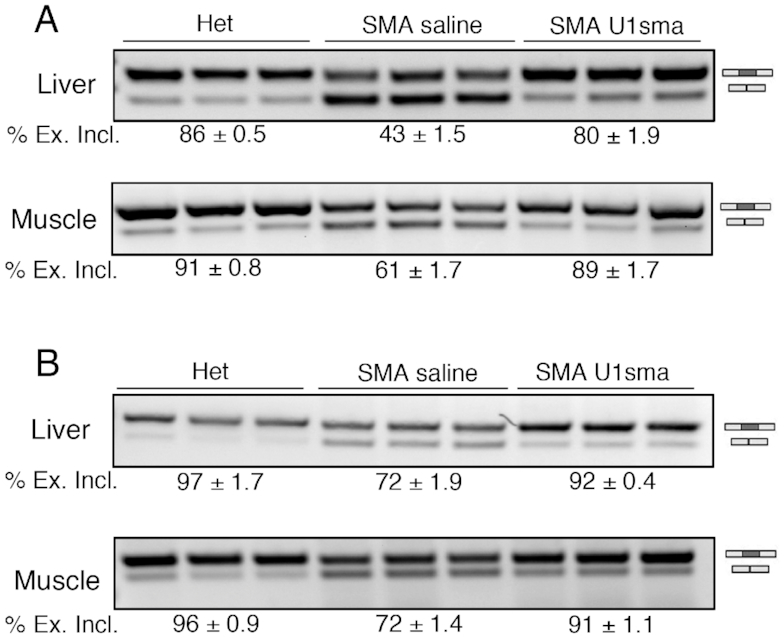
AAV9-ExspeU1sma rescues SMA-induced Mdm2 and Mdm4 alternative splicing defect in severe SMA mice. RT-PCR analysis of (**A**) Mdm2 exon 3 and (**B**) Mdm4 exon 7 splicing pattern in liver and muscle of severe SMA mice treated with AAV9-ExspeU1sma (two intraperitoneal injections, P0 and P2, at 1.5 × 10^12^ vgp/mouse/injection, *n* = 3) and saline (*n* = 3), and of control heterozygous mice treated with saline (*n* = 3) at P7. Endpoint PCR was performed as described (21). Identity of exon inclusion and exclusion bands are indicated. Data are expressed as mean ± SEM of three independent experiments.

Consistent with our AAV-ExspeU1 therapeutic rescue, Comparison Analysis within the Ingenuity software showed a significant rescue of the SMA-deregulated pathways in both liver and muscle of the treated animals. In muscle, the treatment rescued the pathways associated with cell-cycle division and DNA damage response whereas in liver those associated with inflammation (Figure 7A and B). The rescue of DNA damage and cell cycle control pathways is confirmed by RT-PCR analysis of two target skipping exons in *Mdm2* and *Mdm4* previously associated to p53 activation (23). Mdm2 exon 3 was one of the top differentially SE in muscle and liver (Supplementary Data Files S3 and 8, respectively) and the AAV9-ExspeU1sma treatment corrects the SMA-associated Mdm2 and Mdm4 alternative splicing events (Figure 8).

All together these results indicate that AAV9-ExspeU1sma rescues most SMA-induced changes in gene expression and alternative splicing and that this restores deregulated pathways at the systems level in liver and muscle.

### ExspeU1s have few if any off-targets on the human transcriptome

**Figure 9.**
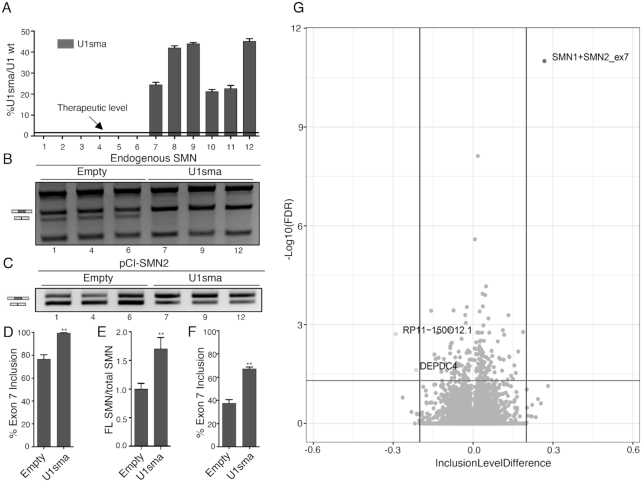
Effects of ExspeU1sma on the human transcriptome. (**A**) ExspeU1sma expression level in HEK 293 Flp-In T-REx stable control cells (lanes 1–6) and stable clone cell line expressing ExspeU1sma (lanes 7–12). The threshold therapeutic level of ExspeU1sma is indicated. Data are expressed as mean ± SD. (**B**) ExspeU1sma rescues endogenous SMN exon 7 inclusion. The indicated clones were analyzed by RT-PCR and amplified fragments digested with *DdeI* restriction enzyme to obtain SMN1 and SMN2 exon 7 inclusion (FL) and exclusion (Δ7) fragments, respectively. The SMN2 exon 7 inclusion and exclusion bands are indicated. (**C**) Transfection experiment of pCI-SMN2 minigene in HEK 293 Flp-In T-REx empty and stable clones. The SMN2 exon 7 inclusion and exclusion bands are indicated. (**D**) Quantification of the percentage of SMN2 exon 7 inclusion of endogenous SMN shown in panel B. (**E**) qPCR of endogenous full length (FL) SMN transcripts to total SMN transcripts in three representative ExspeU1sma stable clones. Expression level of control clones is set to 1. (**F**) Quantification of percentage of SMN2 exon 7 inclusion of pCI-SMN2 minigene transfection experiment shown in panel C. (**G**) Volcano plot showing changes in SE alternative splicing category between empty clones (*n* = 6) and ExspeU1sma stable clones (*n* = 6). Horizontal and vertical lines indicate cut-off values. Dots represent SMN1 + SMN2 exon 7 (ID = 93529), RP11-150O12.1 (ID = 106222) and DEPDC4 (ID = 130674) splicing events (FDR < 0.05; Inclusion Level Difference ≤−0.2 or ≥ 0.2). Significantly affected genes are indicated. In panel (D), (E) and (F) data are expressed as mean ± SD and statistical analysis was performed using Student’s *t*-test (**P < 0.01).

ExspeU1sma off-target pre-mRNA binding sites could cause detrimental changes in gene expression and/or alternative splicing. To evaluate this, we tested the effects of our potential drug on the human transcriptome in non SMN-deficient cells. For this, we created several stable HEK293 Flp-In T-REx clones that contain three integrated copies of *ExspeU1sma* at the same chromosomal location. These cells hugely overexpress ExspeU1sma RNA at between 20 and 40% of wild-type U1 (Figure 9A). This is at least 66 times higher than the putative therapeutically effective levels in SMA animals. As expected ExspeU1sma expression improved endogenous SMN2 exon 7 splicing pattern (Figure 9B and D) and full length SMN RNA (Figure 9E) as well as the minigene SMN2 splicing pattern (Figure 9C and F). This high ExspeU1 expression was completely benign and we found only one differentially expressed gene, BAAT, with the least stringent criteria (no filter on mean counts, log_2_Fold Change ≤−1 or ≥ +1, padj <0.05) between six clones and controls (Supplementary Data File S23). Analysis of the differentially alternative splicing events identified only two alternative exons, other than the intended target SMN exon 7, in *DEPDC4* and *RP11-150O12*. These two genes were far less affected than the SMN target implying that off target splicing effects are negligible (Figure 9G). The improvement in SMN exon 7 is particularly significant and robust (FDR = 0) despite the fact that the counts are the average of both SMN1 and SMN2 transcripts (SMN1 and SMN2 mRNAs have very few sequence differences and SMN1 exon 7 is mostly included). No significant changes were observed for retained introns, alternative splice sites, or mutually excluded exons (Supplementary Data File S24–28). Collectively, these results indicate that ExspeU1sma delivered by AAV9 efficiently recovers the SMA mice and when expressed largely above its therapeutic level targets the human transcriptome with extremely high specificity promoting a robust inclusion of the SMN2 exon 7 without inducing unintended changes in gene expression or alternative splicing.

## DISCUSSION

We show here that the AAV9-mediated expression of a modified U1 core spliceosomal particle is a safe and effective therapeutic strategy for SMA, in two relevant mouse models. This is the first demonstration in animals of a robust therapeutic effect on defective splicing of an AAV vector coding for a modified core spliceosomal component. Systemic treatment of AAV9-ExspeU1sma induces a complete rescue of the tail necrosis in a mild SMA model and a long-term survival in a severe SMA animal with complete recovery of neuromuscular function. The extent of phenotypic improvements induced by systemic treatment of AAV9-ExspeU1sma are comparable with those previously achieved with the antisense oligonucleotide in both animals (15,32). With the AAV delivery system, we showed that the heart and muscle post mitotic transduced tissues had a long-lasting expression of ExspeU1sma above the therapeutic levels and rescue of the SMN2 splicing. This suggests that, in contrast to the several rounds of intratechal injections required for the current approved antisense oligonucleotide therapy, viral delivery of ExspeU1sma would likely maintain therapeutic levels to provide long lasting splicing correction from a single injection.

In general, we observed that tissues with the highest AAV9 DNA also have high expression levels of ExspeU1sma with a parallel correction of SMN2 splicing and SMN protein (Figures 3 and 5). This indicates that the AAV9-ExspeU1sma, with its very short 600 base pair cassette, drives expression of the therapeutic small RNA. Only kidney had high AAV9 viral DNA content but no ExspeU1sma expression nor consistent SMN2 splicing improvement. A lack of correlation was also observed in kidney for the antisense oligonucleotide and might be due to organ peculiarity that accumulates the therapeutic molecules extracellularly (15).

To reach the ExspeU1sma therapeutic range *in vivo* we used a relatively high nominal AAV9 titer. However, due to batch to batch variations and type of preparation (insect vs transfection in mammalian cells) the titer might not reflect the real infectivity of the viral particle *in vivo* (54). Indeed, we noticed that some AAV9-ExspeU1 preparations in mammalian cells can be at least 100 times more efficient in expressing ExspeU1 RNA (data not shown). Future studies should identify the most effective preparation that reaches *in vivo* the therapeutic ExspeU1sma RNA levels in target tissues at the lowest titer.

SMN has been reported to have a DNA repair related function in cellular models (55,56) and DNA damage/repair pathways has been recently reported to be activated in spinal cord (21,22), motoneurons (24) and heart (12) post-mitotic tissues in SMA mice as well as in SMN-depleted cells (22,23). Similarly, activation of inflammatory pathways in liver of SMA animals have been recently proposed to participate in the disease pathogenesis (11,21). We show here that SMN deficiency activates the DNA damage/repair and inflammatory pathways in muscle and liver, respectively, and that AAV9-ExspeU1sma corrects these transcriptome alterations.

Therapeutic strategies based on hybridization to complementary target sequences, may have off-target effects due to their unintended binding to RNAs but these potential side-effects on the transcriptome are infrequently analyzed in detail. Using a cellular model that express ExspeU1sma largely above the therapeutic level we found no relevant undesired effects on the transcriptome nor a general inhibitory effect on pre-mRNA processing. These results are very interesting and promising as this is the first time that a modified core spliceosomal particle is shown to have a high specificity for the intended pre-mRNA with no significant off targets on the human transcriptome. The positive results we obtained on the human transcriptome in HEK293 Flp-In T-REx stable clones should be confirmed in other disease-relevant cells like SMA human induced pluripotent stem cells (iPSCs) differentiated into motor neurons. Motoneurons are considered key target cells for therapy and an ExspeU1sma has been shown to upregulate the SMN protein and improve the motor neurons survival in SMA-iPSCs (57). The extent of phenotypic improvements induced by systemic treatment of AAV9-ExspeU1sma, along with the lack of significant off-target effects on the human transcriptome, are promising for the application of AAV9-ExspeU1sma in the clinic. However, given the long effect we observed *in vivo*, careful preclinical safety studies with different delivery routes will need to be carried out for an extended period of time before planning the advancement into human studies.

Our results are relevant for other diseases caused by exons skipping mutations. We have already shown in cellular systems and/or in asymptomatic mouse models that ExspeU1s correct underlying splicing and protein defects in several disorders (35,36,38,39). Their pronounced efficacy on SMA indicates that these class of molecules have a wide therapeutic potential in many diseases. In addition, viral delivery of ExspeU1 aimed at modulating constitutive alternative exons, in particular at inducing exon inclusion, might be a useful tool for understanding the functional role of normal alternative splicing *in vivo* both in basic research and in splicing therapeutics.

In conclusion, the high efficacy in two SMA animal models, the lack of off-targets effects on the human transcriptome and the long-lasting effect on post-mitotic tissue indicates that U1-modified core spliceosomal particles delivered by AAV might be an additional valid therapeutic opportunity for SMA that can be extended to other splicing pathologies.

## DATA AVAILABILITY

RNA-Seq data have been deposited in Sequence Read Archive (SRA) under accession code PRJNA515903 and PRJNA516147.

## Supplementary Material

gkz469_Supplemental_Files
