# Supplementary material for: Rescue of spinal muscular atrophy mouse models with AAV9-Exon-specific U1 snRNA
Source: Nucleic Acids Res. 2019 May 25;47(14):7618–32. doi: 10.1093/nar/gkz469 (PMC6698663; doi:10.1093/nar/gkz469)
Supplement: gkz469_Supplemental_Files [file gkz469_supplemental_files.zip › Supplementary materials.pdf]

Rescue of Spinal Muscular Atrophy mouse models

by AAV9-Exon Specific U1 snRNA

Irving Donadon[1], Erica Bussani[1], Federico Riccardi[1], Danilo Licastro[2], Giulia Romano[1], Giulia Pianigiani[1], Mirko Pinotti[3], Pavlina Kostantinova[4], Melvin Evers[4], Shuo Lin[5], Markus Rüegg[5] and Franco Pagani[1,*]

[1]Human Molecular Genetics, International Centre for Genetic Engineering and Biotechnology, Padriciano 99, 34149 Trieste, Italy, [2]CBM S.c.r.l., Area Science Park, 34149 Basovizza, Trieste, Italy, [3]Department of Life Sciences and Biotechnology, University of Ferrara, Ferrara, Italy, [4]Department of Research & Development, uniQure biopharma B.V., Amsterdam, The Netherlands, [5]Biozentrum, University of Basel, Klingelbergstrasse 70, 4056 Basel, Switzerland.

* Correspondence: pagani@icgeb.org

Figure Legend

Fig. S1. Base pairing of Exspeu1sma with the SMN2 exon 7 5'ss region. The dark box represents the exon. The sequence of the modified tail of ExspeU1sma and the target sequence on pre-mRNA are indicated. The hnRNPA1-dependent intronic splicing silencer N1 spanning from position +10 to +24 (ISS-N1) is shown.

Fig. S2. Neuromuscular junction and motor neuron analysis in SMA severe-treated animals. (**A**) Representative confocal images of neuromuscular junction (NMJ) of the longissimus capitis and (**B**) schematic representation of α-motor neuron count in severe SMA mice treated with AAV9-U1sma and heterozygous mice treated with saline at P36. Statistical analysis was performed using Student t test (ns, not significant).

Fig. S3. AAV9-ExspeU1sma rescues SMN2 splicing in SMA mild mice. Endpoint PCR of SMN2 splicing pattern in SMA mild ($smn^{-/-}$, $SMN2^{2TG/2TG}$) animals treated with saline (n=3), AAV9-U1wt (n=3) and AAV9-ExspeU1sma (n=3). The upper band of 505 bp corresponds to transcripts including the exon 7; the lower band of 451 bp corresponds to exon skipping.

Fig. S4. AAV9-ExspeU1sma rescues SMN2 splicing in SMA severe mice. (**A**) Endpoint PCR of SMN2 splicing pattern in SMA severe ($smn^{-/-}$, $SMN2^{2TG/0}$) animals treated with saline (n=3; P7), AAV9-U1wt (n=3; P7) and AAV9-ExspeU1sma (n=3; P7, P36, P90 and P160). The upper band of 505 bp corresponds to transcripts including the exon 7; the lower band of 451 bp corresponds to exon skipping. (**B**) Endpoint PCR of SMN2 splicing isoforms in SMA mild animals treated with saline and AAV9-U1wt at P7. Data are expressed as percentage of exon 7 inclusion and statistical analysis was performed using Two Way ANOVA (ns, not significant).

Fig. S5. AAV9-ExspeU1sma rescues SMN protein in severe SMA mice. Western blot analysis of SMN protein in several tissues of severe SMA mice treated with either saline

(n=3; lines 1-3) or AAV9-ExspeU1sma (n=3; lines 4-6) and of heterozygous mice treated with saline (n=2; lines 7-8) and at P7. GAPDH was used as internal reference control. Data quantification is shown in figure 4.

Fig. S6. Alternative splicing events in liver and muscle in SMA severe mice. Fraction of each category of Gencode-annotated splicing events showing increased or decreased alternative isoform use across (**A**) liver and (**B**) muscle in SMA severe ($smn^{-/-}$, $SMN2^{2TG/0}$) mice compared to heterozygous ($smn^{+/-}$, $SMN2^{2TG/0}$) mice treated with saline (FDR <0.05; Inclusion Level Difference ≤ -0.2 or ≥ 0.2).

Fig. S7. Top enriched canonical pathways in liver and muscle of heterozygous versus SMA severe mice. Ingenuity Pathway Analysis (IPA) shows (**A**) in muscle an induction of cell cycle division and DNA damage response pathways, whereas (**B**) in liver pathways linked to inflammation are inducted. Benjamini-Hochberg corrected P value <0.05; activation/repression absolute z-score > 1.5.

Data file S1. Differentially expressed genes in liver of heterozygous versus severe SMA mice.
Data file S2. Differentially expressed genes in muscle of heterozygous versus severe SMA mice.
Data file S3. Splicing changes in Skipped Exon category in muscle of severe SMA versus heterozygous mice.
Data file S4. Splicing changes in Retained intron category in muscle of severe SMA versus heterozygous mice.
Data file S5. Splicing changes in Mutually Exclusive Exons category in muscle of severe SMA versus heterozygous mice.
Data file S6. Splicing changes in Alternative 5' splice sites category in muscle of severe SMA versus heterozygous mice.
Data file S7. Splicing changes in Alternative 3' splice sites category in muscle of severe SMA versus heterozygous mice.
Data file S8. Splicing changes in Skipped Exon category in liver of severe SMA versus heterozygous mice.
Data file S9. Splicing changes in Retained intron category in liver of severe SMA versus heterozygous mice.
Data file S10. Splicing changes in Mutually Exclusive Exons category in liver of severe SMA versus heterozygous mice.
Data file S11. Splicing changes in Alternative 5' splice sites category in liver of severe SMA versus heterozygous mice.
Data file S12. Splicing changes in Alternative 3' splice sites category in liver of severe SMA versus heterozygous mice.
Data file S13. Splicing changes in Skipped Exon category in muscle of severe SMA vs AAV9-ExspeU1sma treated mice.
Data file S14. Splicing changes in Retained intron category in muscle of severe SMA AAV9-ExspeU1sma treated mice.
Data file S15. Splicing changes in Mutually Exclusive Exons category in muscle of severe SMA vs AAV9-ExspeU1sma treated mice.
Data file S16. Splicing changes in Alternative 5' splice sites category in muscle of severe SMA vs AAV9-ExspeU1sma treated mice.

Data file S17. Splicing changes in Alternative 3' splice sites category in muscle of severe SMA vs AAV9-ExspeU1sma treated mice.
Data file S18. Splicing changes in Skipped Exon category in liver of severe SMA vs AAV9-ExspeU1sma treated mice.
Data file S19. Splicing changes in Retained intron category in liver of severe SMA vs AAV9-ExspeU1sma treated mice.
Data file S20. Splicing changes in Mutually Exclusive Exons category in liver of severe SMA vs AAV9-ExspeU1sma treated mice.
Data file S21. Splicing changes in Alternative 5' splice sites category in liver of severe SMA vs AAV9-ExspeU1sma treated mice.
Data file S22. Splicing changes in Alternative 3' splice sites category in liver of severe SMA vs AAV9-ExspeU1sma treated mice.
Data file S23. Differentially expressed genes in empty versus ExspeU1sma stable clones.
Data file S24. Splicing changes in Skipped Exon category in empty versus ExspeU1sma stable clones.
Data file S25. Splicing changes in Retained intron category in empty versus ExspeU1sma stable clones.
Data file S26. Splicing changes in Mutually Exclusive Exons category in empty versus ExspeU1sma stable clones.
Data file S27. Splicing changes in Alternative 5' Splice Sites category in empty versus ExspeU1sma stable clones.
Data file S28. Splicing changes in Alternative 3' Splice Sites category in empty versus ExspeU1sma stable clones.
Data file S29. Significantly differentially expressed genes in liver and muscle of heterozygous versus severe SMA mice.
Data file S30. Significantly splicing changes in alternative splicing categories (SE, RI, MXE, A5SS and A3SS) in liver and muscle of heterozygous versus severe SMA mice.

Table S1. List of primers used in this study.

Fig S1

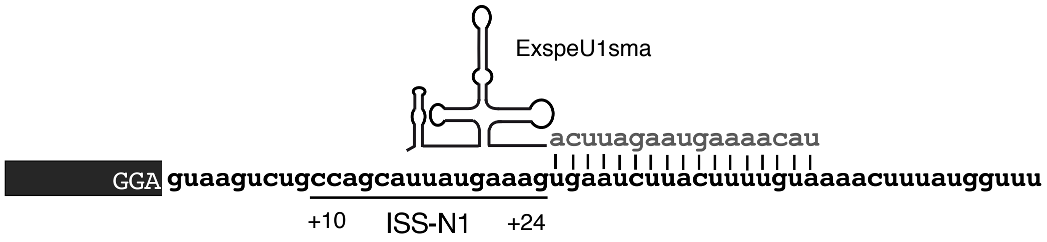
ExspeU1sma
acuuagaaugaaaacau
GGA guaagucugccagcauuaugaaagugaaucuuacuuuuguaaaacuuuaugguuu
+10
ISS-N1
+24

Figure S2

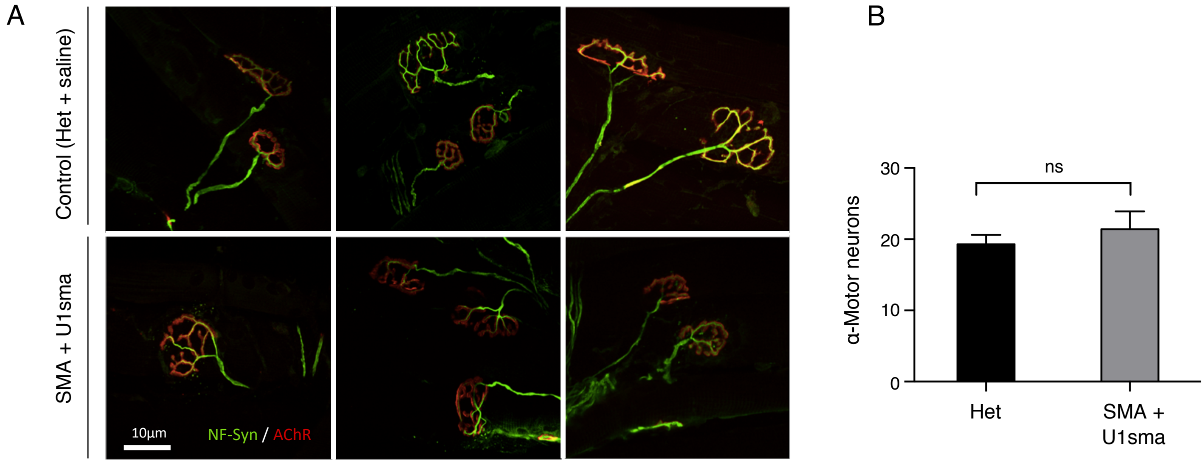
A
Control (Het + saline)
SMA + U1sma
10µm
NF-Syn / AChR
B
α-Motor neurons
30
20
10
0
ns
Het
SMA +
U1sma

Figure S3

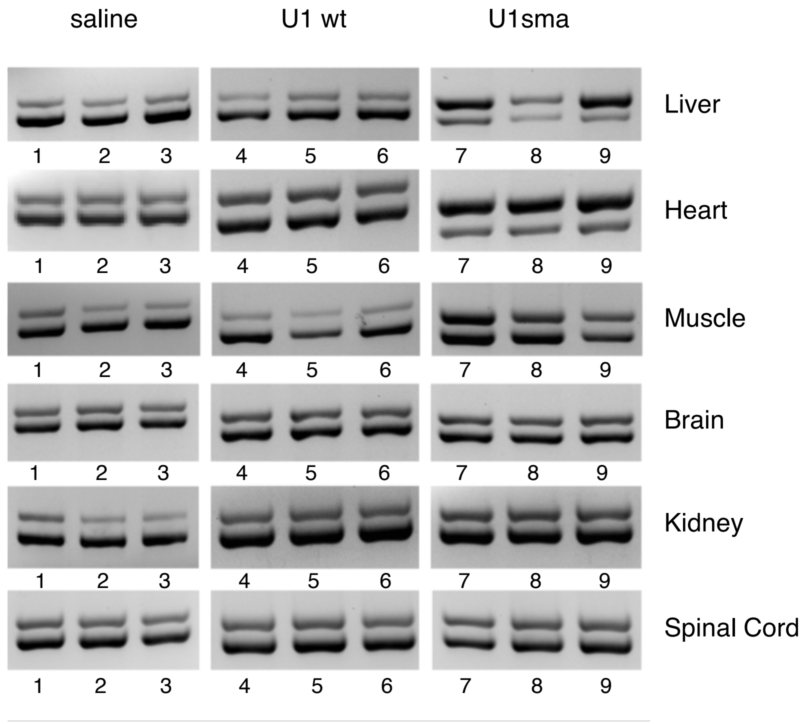
saline
U1 wt
U1sma
Liver
Heart
Muscle
Brain
Kidney
Spinal Cord
1 2 3
4 5 6
7 8 9

Figure S4

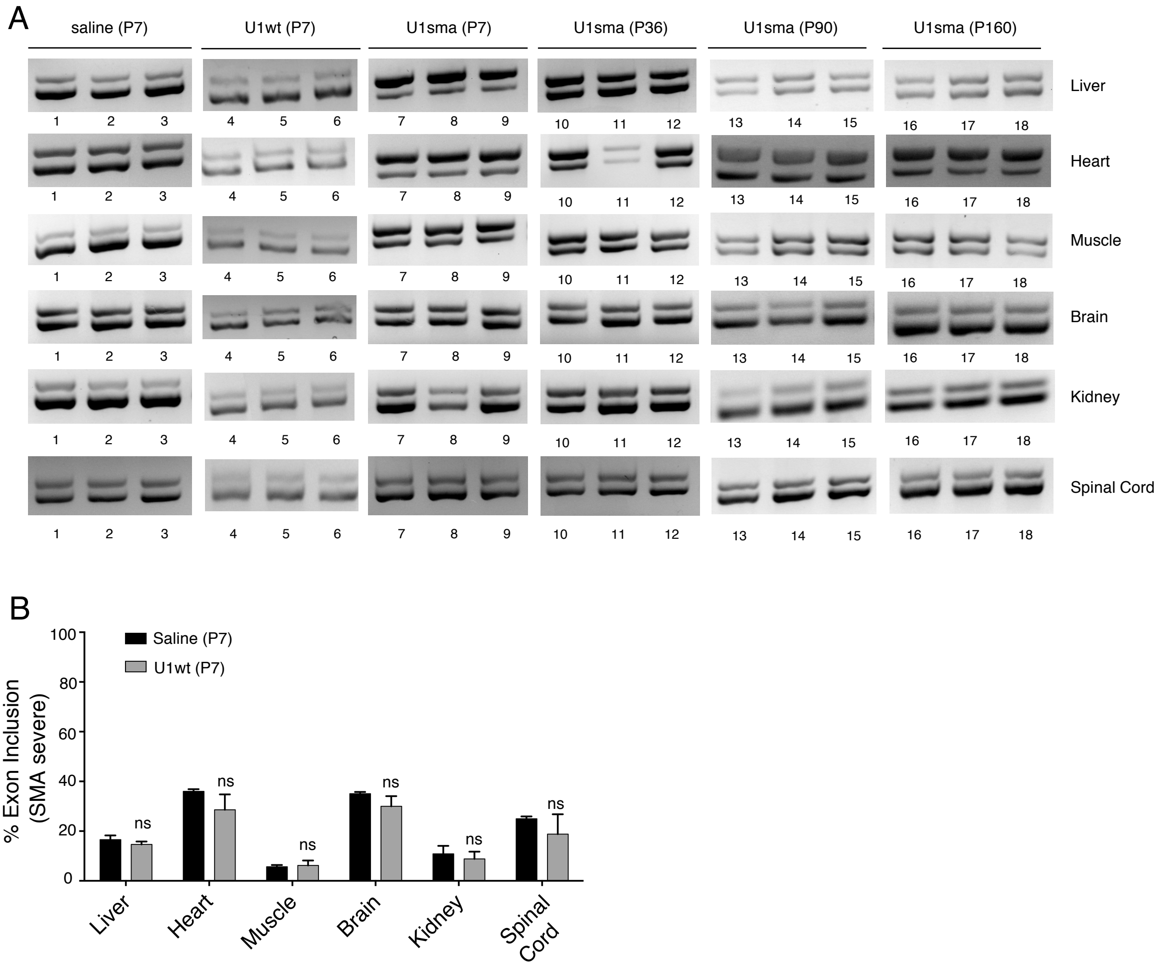
A
saline (P7)
U1wt (P7)
U1sma (P7)
U1sma (P36)
U1sma (P90)
U1sma (P160)
Liver
Heart
Muscle
Brain
Kidney
Spinal Cord
1 2 3 4 5 6 7 8 9 10 11 12 13 14 15 16 17 18
B
Saline (P7)
U1wt (P7)
% Exon Inclusion (SMA severe)
100
80
60
40
20
0
ns
Liver
Heart
Muscle
Brain
Kidney
Spinal Cord

Figure S5

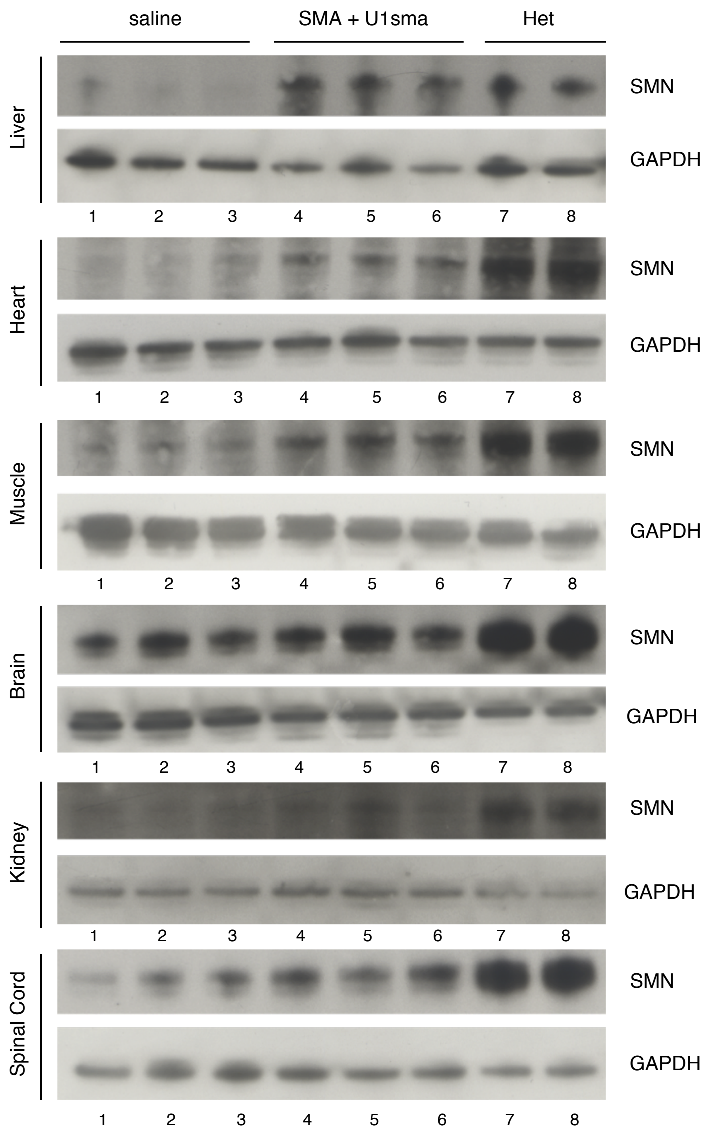
saline
SMA + U1sma
Het
Liver
Heart
Muscle
Brain
Kidney
Spinal Cord
SMN
GAPDH
1 2 3 4 5 6 7 8

Figure S6

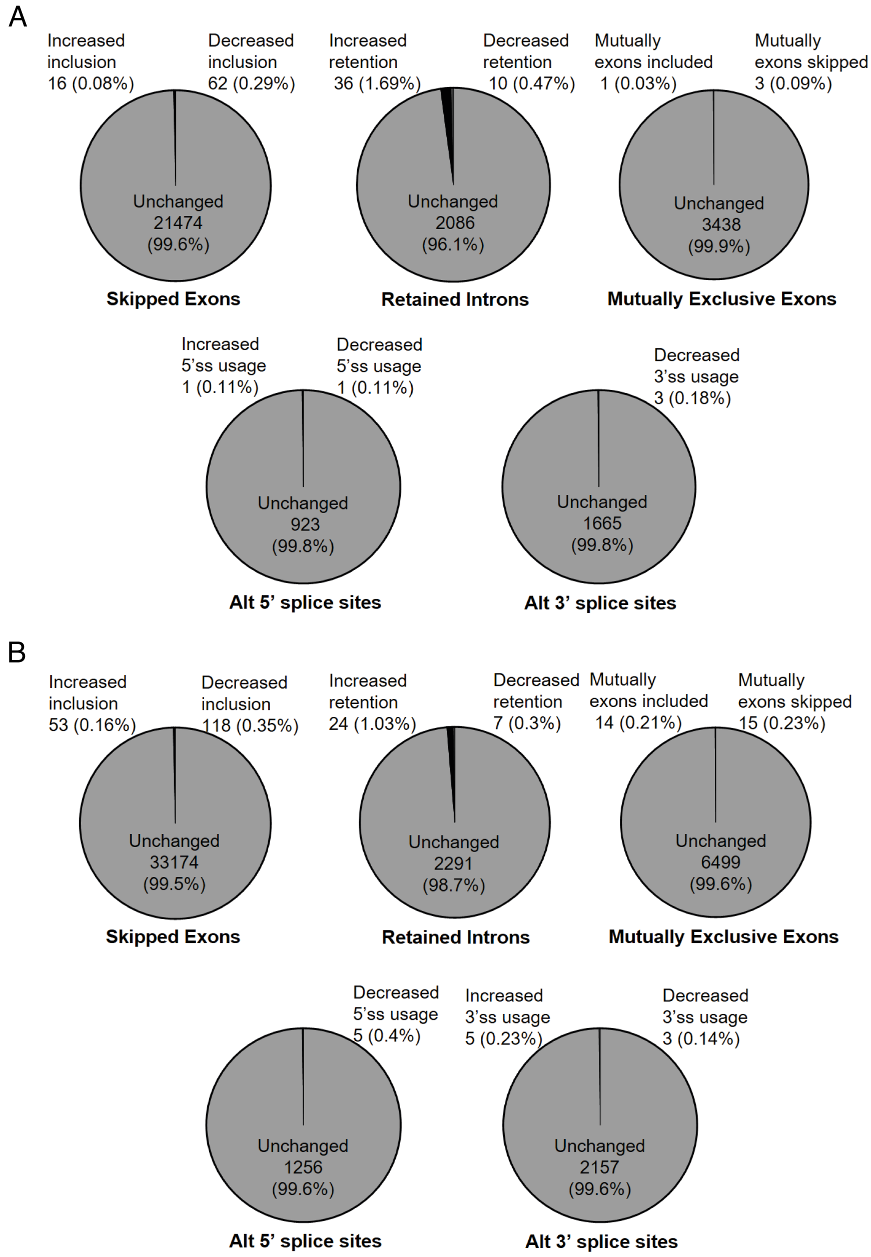
A
Increased inclusion 16 (0.08%)
Decreased inclusion 62 (0.29%)
Unchanged 21474 (99.6%)
Skipped Exons
Increased retention 36 (1.69%)
Decreased retention 10 (0.47%)
Unchanged 2086 (96.1%)
Retained Introns
Mutually exons included 1 (0.03%)
Mutually exons skipped 3 (0.09%)
Unchanged 3438 (99.9%)
Mutually Exclusive Exons
Increased 5'ss usage 1 (0.11%)
Decreased 5'ss usage 1 (0.11%)
Unchanged 923 (99.8%)
Alt 5' splice sites
Decreased 3'ss usage 3 (0.18%)
Unchanged 1665 (99.8%)
Alt 3' splice sites
B
Increased inclusion 53 (0.16%)
Decreased inclusion 118 (0.35%)
Unchanged 33174 (99.5%)
Skipped Exons
Increased retention 24 (1.03%)
Decreased retention 7 (0.3%)
Unchanged 2291 (98.7%)
Retained Introns
Mutually exons included 14 (0.21%)
Mutually exons skipped 15 (0.23%)
Unchanged 6499 (99.6%)
Mutually Exclusive Exons
Decreased 5'ss usage 5 (0.4%)
Unchanged 1256 (99.6%)
Alt 5' splice sites
Increased 3'ss usage 5 (0.23%)
Decreased 3'ss usage 3 (0.14%)
Unchanged 2157 (99.6%)
Alt 3' splice sites

Figure S7

A

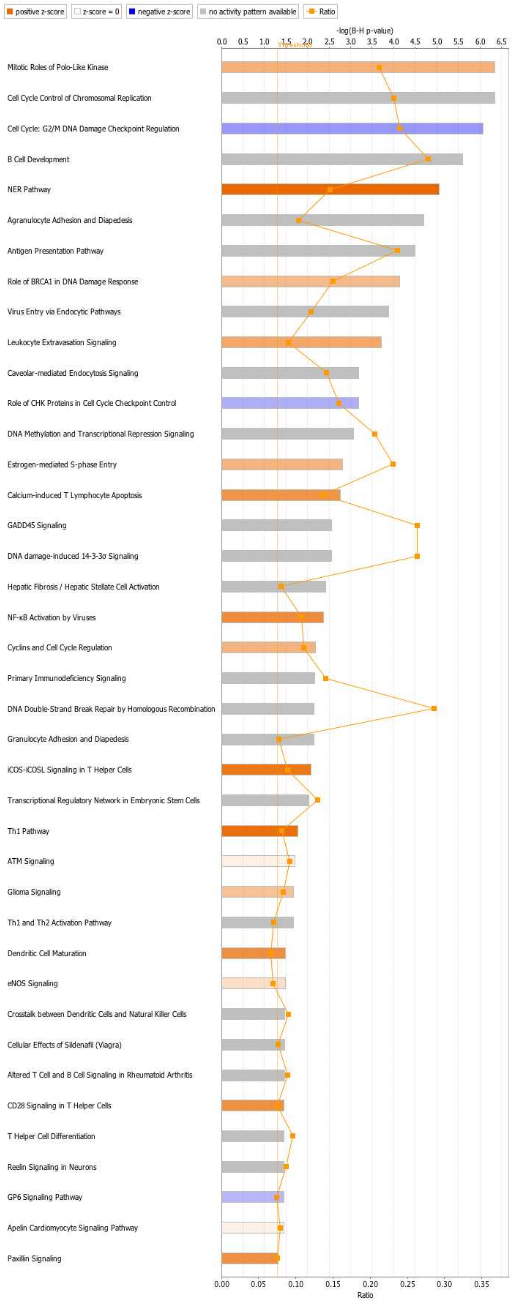
positive z-score
z-score = 0
negative z-score
no activity pattern available
Ratio
-log(B-H p-value)
Mitotic Roles of Polo-Like Kinase
Cell Cycle Control of Chromosomal Replication
Cell Cycle: G2/M DNA Damage Checkpoint Regulation
B Cell Development
NER Pathway
Agranulocyte Adhesion and Diapedesis
Antigen Presentation Pathway
Role of BRCA1 in DNA Damage Response
Virus Entry via Endocytic Pathways
Leukocyte Extravasation Signaling
Caveolar-mediated Endocytosis Signaling
Role of CHK Proteins in Cell Cycle Checkpoint Control
DNA Methylation and Transcriptional Repression Signaling
Estrogen-mediated S-phase Entry
Calcium-induced T Lymphocyte Apoptosis
GADD45 Signaling
DNA damage-induced 14-3-3σ Signaling
Hepatic Fibrosis / Hepatic Stellate Cell Activation
NF-κB Activation by Viruses
Cyclins and Cell Cycle Regulation
Primary Immunodeficiency Signaling
DNA Double-Strand Break Repair by Homologous Recombination
Granulocyte Adhesion and Diapedesis
iCOS-iCOSL Signaling in T Helper Cells
Transcriptional Regulatory Network in Embryonic Stem Cells
Th1 Pathway
ATM Signaling
Glioma Signaling
Th1 and Th2 Activation Pathway
Dendritic Cell Maturation
eNOS Signaling
Crosstalk between Dendritic Cells and Natural Killer Cells
Cellular Effects of Sildenafil (Viagra)
Altered T Cell and B Cell Signaling in Rheumatoid Arthritis
CD28 Signaling in T Helper Cells
T Helper Cell Differentiation
Reelin Signaling in Neurons
GP6 Signaling Pathway
Apelin Cardiomyocyte Signaling Pathway
Paxillin Signaling
Ratio

B

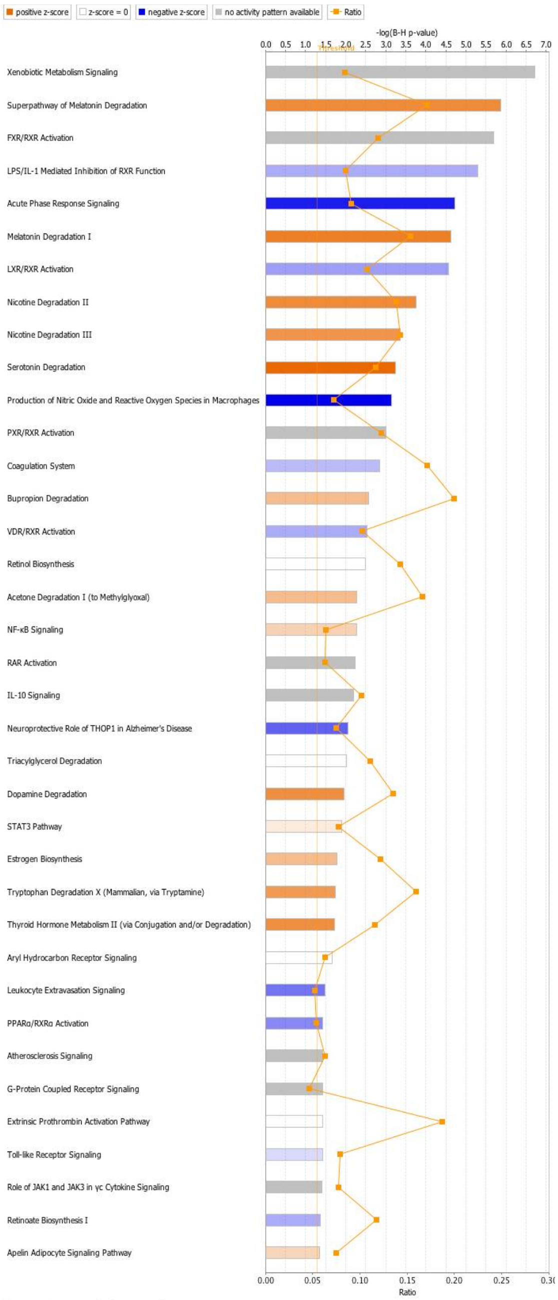
positive z-score
z-score = 0
negative z-score
no activity pattern available
Ratio
-log(B-H p-value)
Xenobiotic Metabolism Signaling
Superpathway of Melatonin Degradation
FXR/RXR Activation
LPS/IL-1 Mediated Inhibition of RXR Function
Acute Phase Response Signaling
Melatonin Degradation I
LXR/RXR Activation
Nicotine Degradation II
Nicotine Degradation III
Serotonin Degradation
Production of Nitric Oxide and Reactive Oxygen Species in Macrophages
PXR/RXR Activation
Coagulation System
Bupropion Degradation
VDR/RXR Activation
Retinol Biosynthesis
Acetone Degradation I (to Methylglyoxal)
NF-κB Signaling
RAR Activation
IL-10 Signaling
Neuroprotective Role of THOP1 in Alzheimer's Disease
Triacylglycerol Degradation
Dopamine Degradation
STAT3 Pathway
Estrogen Biosynthesis
Tryptophan Degradation X (Mammalian, via Tryptamine)
Thyroid Hormone Metabolism II (via Conjugation and/or Degradation)
Aryl Hydrocarbon Receptor Signaling
Leukocyte Extravasation Signaling
PPARα/RXRα Activation
Atherosclerosis Signaling
G-Protein Coupled Receptor Signaling
Extrinsic Prothrombin Activation Pathway
Toll-like Receptor Signaling
Role of JAK1 and JAK3 in γc Cytokine Signaling
Retinoate Biosynthesis I
Apelin Adipocyte Signaling Pathway
Ratio

Table S1

Primer's name	Primers' sequence
U1 WT Fw A	5’-ATACTTACCTGGCAGGGGAGAT-3’
SM25 Fw New_2	5’-TACAAAAGTAAGATTCAGCAG-3’
U1 160 Rev_2	5’-GGAAAGCGCGAACGCAGTCCCCCAC-3’
pCI Fwd B	5’-GACTCACTATAGGCTAGCCT-3’
E8 - 75 + 5’Rev	5’-CTGTCTAGACCTTACACTTCGCAATGTCCATTCATGAA-3’
E6 Fw	5’-ATAATTCCCCCACCACCTCCC-3’
E8-467 Rev	5’-TTGCCACATACGCCTCACATAC-3’
qGAPDH Fw	5’-GACAGTCAGCCGCATCTTCT-3’
qGAPDH Rev	5’-TTAAAAGCAGCCCTGGTGAC-3’
mGAPDH Fw	5’-ATGGTGAAGGTCGGTGTGAA-3’
mGAPDH Rev	5’-GTTGATGGCAACAATCTCCA-3’
Ex7 Fw	5’-GCTGATGCTTTGGGAAGTATGTTA-3'
Ex8 Rev	5'-CACCTTCCTTCTTTTTGATTTTGTC-3'
Ex2a Fw	5’-AGCGATGATTCTGACATTTGGGATG-3’
Ex2b Rev	5’-CTGTTGTAAGGAAGCTGCAGTATTCTT-3’
U1WT FW2	5'-TCTTTACACACACGG-3'
U1WT RV2	5'-GAGGGAAAAAGGGAG-3'
U1WT ITR FW	5'-GGTTCCTCAGATCGATCC-3'
U1WT U1 ter RV	5'-TTTCTGGAGTTTCAAAAGTAGA-3'
SMN2 FL DIR	5’-CAAAAAGAAGGAAGGTGCTCACATT-3’
SMN2 REV C	5’-GCTTCACATTCCAGATCTGTC-3’
